# Enhancing Connected Health Ecosystems Through IoT-Enabled Monitoring Technologies: A Case Study of the Monit4Healthy System

**DOI:** 10.3390/s25072292

**Published:** 2025-04-04

**Authors:** Marilena Ianculescu, Victor-Ștefan Constantin, Andreea-Maria Gușatu, Mihail-Cristian Petrache, Alina-Georgiana Mihăescu, Ovidiu Bica, Adriana Alexandru

**Affiliations:** 1 National Institute for Research and Development in Informatics, 011455 Bucharest, Romania; marilena.ianculescu@ici.ro (M.I.); cristian.petrache@ici.ro (M.-C.P.); alina.mihaescu@ici.ro (A.-G.M.); ovidiu.bica@ici.ro (O.B.); adriana.alexandru@ici.ro (A.A.); 2 Faculty of Electrical Engineering, Electronics and Information Technology, Valahia University of Targoviste, 130004 Targoviste, Romania

**Keywords:** IoT, connected health ecosystems, health monitoring, Monit4Healthy system, sensor fusion, real-time biomedical signal processing

## Abstract

The Monit4Healthy system is an IoT-enabled health monitoring solution designed to address critical challenges in real-time biomedical signal processing, energy efficiency, and data transmission. The system’s modular design merges wireless communication components alongside a number of physiological sensors, including galvanic skin response, electromyography, photoplethysmography, and EKG, to allow for the remote gathering and evaluation of health information. In order to decrease network load and enable the quick identification of abnormalities, edge computing is used for real-time signal filtering and feature extraction. Flexible data transmission based on context and available bandwidth is provided through a hybrid communication approach that includes Bluetooth Low Energy and Wi-Fi. Under typical monitoring scenarios, laboratory testing shows reliable wireless connectivity and ongoing battery-powered operation. The Monit4Healthy system is appropriate for scalable deployment in connected health ecosystems and portable health monitoring due to its responsive power management approaches and structured data transmission, which improve the resiliency of the system. The system ensures the reliability of signals whilst lowering latency and data volume in comparison to conventional cloud-only systems. Limitations include the requirement for energy profiling, distinctive hardware miniaturizing, and sustained real-world validation. By integrating context-aware processing, flexible design, and effective communication, the Monit4Healthy system complements existing IoT health solutions and promotes better integration in clinical and smart city healthcare environments.

## 1. Introduction

By enabling highly connected, real-time health monitoring ecosystems featuring the use of biomedical sensors, cutting-edge communication technologies, and complex data processing frameworks, the Internet of Things (IoT) has totally reshaped the world of healthcare. The growing demand for ongoing, remote patient monitoring, notably for the management of chronic diseases, rehabilitation from surgeries, and eldercare, is a major driver of this approach toward societal transformation [1]. Traditional healthcare models are changing dramatically in their pursuit of data-centric, preventative, and highly customized medicine. Such models have previously hinged on established in-person consultations and reactive forms of therapy. Real-time patient monitoring is distinguished as an integral part of clinical decision making in this constantly evolving setting, facilitating preventative care and greatly boosting patient outcomes.

Patient care, disease prevention, and comprehensive health management are all influenced by a complex system of healthcare professionals, medical establishments, innovative technologies, and regulatory frameworks that jointly make up a health ecosystem. These ecosystems are typically confined to big healthcare institutions, notably clinics, hospitals, and specialized care centers, where physicians establish diagnoses and manage patients mostly through recurrent relationships with patients and subsequent data analysis [2]. The easy share of clinical information, the merging of interdisciplinary healthcare services, and the accessibility of medical infrastructure that enables both therapeutic and preventive procedures are all key to the effectiveness of such ecosystems. Concerns ranging from fragmented data, inadequate interoperability, and limited access to real-time patient information are prevalent in traditional health ecosystems and can compromise clinical decision making and interfere with the continuity of qualitative care.

By taking advantage of digital transformation, IoT-driven sensor networks, cloud-powered analytics, and artificial intelligence (AI) for providing real-time, data-driven healthcare services, a connected health ecosystem optimizes traditional healthcare setups. This approach allows for the uninterrupted gathering of data, secure transmission, and smart processing throughout different medical contexts by smoothly integrating medical devices, wearable sensors, remote monitoring systems, and electronic health records. In addition to strengthening patient monitoring, this integrated model supports more proactive, tailored, and reliable healthcare delivery. A connected health ecosystem facilitates the systematic detection of abnormalities, the predictive modeling of disease evolution, and continuous remote health monitoring and management, as opposed to traditional health systems where the observation of patients is intermittent and exclusive to hospitals and medical centers.

Through the application of biomedical sensors, edge computing, and wireless communication protocols to provide real-time, automated, and continuous health evaluations, the integration of IoT-enabled monitoring technologies has radically changed the medical field [3]. Such advances enhance patient monitoring capabilities while rendering it more feasible to identify health abnormalities at an early stage as it allows for the straightforward gathering, exchange, and analysis of physiological and physical data [4]. IoT-enabled systems feature the real-time tracking of key health parameters within medical centers, remote healthcare environments, and smart urban infrastructures, in contrast to traditional health monitoring solutions that primarily depend on random assessments conducted at clinical appointments. A more thorough patient monitoring and improved clinical decision support originated from the development of multi-sensor systems that integrate technologies like electrocardiography (ECG), oxygen saturation (SpO_2_), electromyography (EMG), galvanic skin response (GSR), and motion-tracking technologies. Such systems have also significantly boosted the accuracy and extent of digital health assessments. Incorporating edge computing architectures with low-latency communication protocols maximizes data processing efficiency, reduces dependency on centralized cloud computing, and enables the prompt identification of abnormalities and system resilience. In line with rising demands for integrated healthcare ecosystems and efficient smart city integration, these improvements in technology have widened up the possibility for scalable, secure, and energy-efficient health monitoring solutions.

Multi-sensor IoT health monitoring solutions have lately progressed amid notable developments in wireless communication, sensor miniaturization, and real-time analytics. The timely detection of abnormalities has been enhanced by the merging of AI-driven health analytics with real-time physiological data collection, facilitating built-in alerts, predictive analytics, and the recognition of health fluctuations at the outset. By simply integrating IoT-enabled health solutions into telemedicine infrastructures, remote patient health monitoring systems most especially have radically shifted the way healthcare is delivered [5]. They make certain that real-time physiological data are adequately shared and processed in order to facilitate remote clinical evaluations.

Emerging IoT health ecosystems improve personalized care by fostering efficient interaction between patients, healthcare providers, and smart urban health infrastructure. As IoT-enabled technologies develop further, their impact transcends well beyond monitoring a single patient; they could ultimately be applied to guide health policy decisions, streamline hospital resource management, and even deliver population-level smart health oversight on an extensive basis.

IoT-enabled health monitoring is evolving quickly, but there are still a number of important challenges to overcome, especially when it comes to real-time data transmission, sensor communication, system scalability, and strict security standard compliance. For healthcare applications like movement analysis, neuromuscular evaluations, and cardiac analysis that need ultra-low latency and high-performance monitoring in order to provide reliable and helpful data to healthcare professionals, they have to be operated practically in real time. There are compromises among latency, bandwidth capacity, and energy efficiency brought about by frequently implemented wireless communication protocols such as Wi-Fi (Wireless Fidelity) and Bluetooth Low Energy (BLE) [6]. Although Wi-Fi allows for fast data transfer, in high-density healthcare environments, it requires significant amounts of power and can be vulnerable to network bottlenecks. Large-scale healthcare implementations could face limitations due to BLE’s limited bandwidth and inadequate data throughput, despite its low-power operation optimization.

The implementation of low-power, long-range IoT communication technologies, including NB-IoT and Long-Range Wide-Area Network (LoRaWAN), which are increasingly endorsed for having the capacity for establishing scalable remote health monitoring infrastructure [7,8], is an alternative option. These protocols are especially appropriate for the continuous monitoring of patients with increased risk in residence-based care settings because they convey wide-area data transmission with low power consumption. Their slower transfer speeds restrict their ability to gather high-frequency biomedical parameters, exposing a basic compromise between real-time responsiveness and energy efficiency. Adaptive communication models that combine system performance, scalability, and real-time medical insights by boosting data transmission efficiency depending on particular healthcare solution demands need to be developed to tackle this issue.

Achieving flattened multi-sensor integration and interoperability is one of the key hurdles in IoT-based healthcare ecosystems. It remains difficult to manage data fusion, generate effective sensor communication, and maintain shared interoperability since plenty of the current health monitoring systems are based on fragmented and heterogeneous sensor architectures. Scalability and simple integration into current health information systems and urban IoT infrastructures are hampered by large variations in sensor approaches, operating frequencies, and proprietary communication protocols, which add complexity to comprehensive data standards. MQTT-based IoT messaging and HL7 Fast Healthcare Interoperability Resources (FHIRs) are two recent improvements in standardized frameworks that attempt to address these shortcomings in interoperability [9,10], but acceptance is still lagging because of ongoing standardization shortages, existing system limitations, and regulatory complexities.

As IoT-enabled healthcare solutions keep developing, data security and patient privacy become more important challenges transcending interoperability. End-to-end encryption, reliable authentication procedures, and strict compliance with healthcare data protection security like the General Data Protection Regulation (GDPR) and the Health Insurance Portability and Accountability Act (HIPAA) [11] are prerequisites for widespread IoT health solutions. In order to prevent data breaches, unauthorized access, and cyberattacks on critical healthcare infrastructure, robust cybersecurity measures must be implemented given the increasing number of connected medical devices distributing highly sensitive patient data. Scalable and secure data-sharing frameworks are becoming incrementally more significant as IoT health monitoring devices [12] connect more and more with smart city infrastructures. Decentralized health data management systems, encrypted data-sharing protocols, and secure cloud infrastructures must be implemented to tackle these challenges and protect patient data while facilitating real-time, data-driven healthcare delivery.

Recent research efforts have been focused on bolstering real-time processing frameworks, improving data transmission protocols, and expanding sensor-to-network communication in IoT healthcare settings [13,14,15] in order to mitigate such barriers. A key driver in increasing data processing performance, reducing network bottlenecks, and maintaining continuous health monitoring in insightful, application scenarios [16] is the widespread use of versatile IoT communication models that incorporate low-latency transmission protocols and edge computing architectures. The emergence of edge computing architectures in IoT-based healthcare solutions is among the most significant improvements in this domain. Edge computing renders it possible to preprocess data in real time at the sensor or gateway level, as opposed to conventional cloud-centric approaches that are frequently limited by latency and bandwidth issues. It lessens the computational load on centralized healthcare servers while improving transmission efficiency. These solutions might instantly recognize abnormalities, filter redundant data, and trigger alerts by integrating AI-driven signal processing capabilities. This boosts system responsiveness and supports medical decision making.

New opportunities for extensive, real-time health monitoring are being brought about by the convergence of IoT health monitoring with smart city infrastructures. It opens up new opportunities for public health monitoring, emergency response synchronization, and AI-enhanced demographic surveillance when IoT-enabled healthcare solutions are plugged into smart city ecosystems. Real-time predictive analytics for major health events and location-aware health services may be realized through the use of low-power, distributed health monitoring devices that proactively link with urban IoT infrastructures. Such developments strengthen the role of IoT in influencing healthcare’s evolution by fostering the emergence of public health systems that are more robust and responsive.

This paper presents the Monit4Healthy system, an extensive platform that integrates multi-sensor fusion, optimized real-time biomedical signal processing, and effective IoT communication frameworks to improve connected health ecosystems in line with a rising need for proficient IoT-enabled health monitoring solutions. By tackling important concerns with sensor interoperability, low-latency data transmission, and secure health data exchange, the system contributes to developing healthcare infrastructures that are more savvy, versatile, and interconnected.

The Monit4Healthy system features a modular sensor fusion architecture to provide thorough physiological and biomechanical health evaluations, in contrast with other IoT health platforms that frequently focus on collecting single-sensor data. By ensuring consistent data integration, this system promotes both the accuracy and efficacy of health monitoring. By combining edge computing, secure communication protocols, and smart data transmission approaches, the system improves the scalability, efficiency, and reliability of real-time health monitoring solutions. Its structure endorses remote healthcare management and continuous patient monitoring, where all of the above are part of the larger context of next-generation IoT-based connected health ecosystems. The conceptual framework of the system may be customized to fit the health infrastructures of smart cities, enabling population-wide health analytics, emergency response coordination, and real-time health tracking. With the aforementioned approaches, the Monit4Healthy system accounts for the establishment of smart and data-driven healthcare solutions by demonstrating an affordable and reliable way of overcoming current bottlenecks in IoT healthcare solutions.

By merging multi-sensor fusion with a flexible and configurable architecture, the Monit4Healthy system enables a novel approach to IoT-enabled health monitoring that is appropriate for a range of healthcare settings. By providing context-aware physiological and biomechanical monitoring, the Monit4Healthy system helps to guarantee a better degree of accuracy and accessibility in both clinical and non-clinical environments, in contrast to traditional IoT health solutions that typically focus on collecting single-sensor data. Its focus on smart information interchange and real-time physiological signal processing facilitates better decision making in connected health ecosystems. The Monit4Healthy system’s architecture takes into account real-world implementation concerns certifying that the system can be smoothly integrated into current digital health infrastructures with no mandating major architectural changes. In tackling current challenges with interoperability, data accuracy, and secure sensor communication, these characteristics showcase the Monit4Healthy system as a constructive tool for IoT-driven healthcare and boost the position of connected health ecosystems in next-generation healthcare solutions.

Many present solutions are still constrained by fragmented sensor architectures, a lack of real-time edge processing, and ineffective data transmission procedures, which impede scalability, accuracy, and adaptability in actual healthcare environments, even with notable advancements in IoT-based healthcare monitoring. These drawbacks underscore the urgent need for integrated solutions that provide context-aware processing, set up, low-latency communication, and multi-sensor data fusion across adaptable and interoperable technologies. The Monit4Healthy system was designed specifically to meet these gaps by providing a cohesive and flexible approach that overcomes the present drawbacks of IoT health monitoring technology.

Even though several IoT-enabled health monitoring systems have emerged lately, they often face significant shortcomings when it comes to integrating several sensors easily, processing data adaptively, and communicating effectively in real time when resources are limited. Particularly in wearable and portable health monitoring circumstances, high latency, inefficient bandwidth, and increased energy consumption are caused by the inflexible architectures found in several current digital health solutions, such as in cloud-dependent processing and continuous raw data streaming. An extensive number of IoT-based health solutions rely on single-sensor data collection processes and are not sufficiently versatile to provide scaled, context-aware health monitoring in a variety of settings. Section 2, which assesses the current situation of IoT-based health communication technologies and underlines the functional constraints of present systems, provides a detailed analysis of these shortcomings.

In order to ensure low-latency communication and interoperability with current digital health infrastructures, adaptive, modular IoT health systems that can perform multi-sensor fusion, structured data transmission, and edge-level signal processing are required. These challenges highlight a clearly defined research gap. As a way to bridge this gap, the digital health solutions must be able to minimize computational overhead and energy consumption while constantly adjusting gathering and transmission techniques in response to the real-time physiological parameters and limitations of the network.

In an attempt to swiftly tackle these operational and technological issues, the Monit4Healthy system was designed and developed. In particular, in environments that demand trustworthy remote management and compatibility with connected health ecosystems, such as smart city healthcare infrastructures, its modular architecture, context-aware signal processing, and structured, event-driven data transmission framework as a whole comprise an alternative and integrated way of advancing IoT-based health monitoring. Thus, the Monit4Healthy system is a base for future implementations in real-world healthcare and city environments, offering a scalable and adaptable solution to the present drawbacks of IoT health monitoring systems.

This paper’s remaining sections are organized as follows: Section 2 provides a comprehensive review of IoT-based health communication technologies, focusing on advancements in sensor fusion and real-time biomedical signal processing. Section 3 details the Monit4Healthy system architecture, covering sensor integration, communication frameworks, and data acquisition processes. Current results are discussed in Section 4, where the system’s sensor performance, data transmission efficiency, and overall functional reliability are evaluated. The Monit4Healthy system’s broader impacts are covered in Section 5, which also examines its potential to integrate with smart city infrastructures. Lastly, Section 6 highlights the significance of IoT in forming next-generation healthcare solutions while summarizing significant accomplishments and outlining future research areas.

## 2. Literature Review

### 2.1. IoT Technologies in Health Monitoring

Proactive medical interventions, early disease identification, and ongoing remote patient surveillance are made possible by IoT-enabled health monitoring systems. Digital healthcare is changing as a result of the integration of wearable sensors, cloud-based platforms, and AI-driven analytics. By improving operational efficiency, giving medical personnel data-driven insights, and encouraging proactive, personalized treatment, the ongoing development of IoT technologies in healthcare has completely changed how patient care is delivered.

#### 2.1.1. Key Advancements and Applications of IoT in Healthcare

IoT-enabled devices facilitate the 24/7 real-time health monitoring of vital signs like blood pressure, heart rate, and SpO_2_. This enables more effective patient care by facilitating the early detection of medical conditions, improved chronic disease management, and timely alerts for healthcare professionals [17].

Advanced sensors are used by wearable devices like smartwatches, fitness trackers, and biosensors to monitor a variety of parameters, including heart rate and rhythm, ECG, sleep patterns (duration and quality of sleep), activity levels (steps, total movement, motion tracking), physiological signs of stress, etc. Algorithms are used to gather huge amounts of real-time health data [18]. Medical practitioners can take preventative measures for early warning systems for metabolic and cardiovascular disorders.

Remote diagnosis and seamless communication are made possible by integrating IoT devices with telemedicine platforms. This decreases needless hospital stays, increases access to healthcare in underserved and rural areas, and improves senior care through remote monitoring [19].

Large volumes of data are produced by IoT devices, and machine learning and AI are used to integrate and analyze these data. Insights from predictive analytics are utilized to prevent disease, provide personalized treatment plans, and make better operational decisions in hospitals [20].

Real-time patient, staff, and medical equipment tracking is made possible by IoT-enabled infrastructure, which speeds up emergency response times and optimizes resource use in hospital and smart home systems [21].

Key IoT applications in the healthcare sector include the following:

a. Chronic Disease Management—In order to effectively manage chronic conditions, IoT devices like blood pressure monitors, connected inhalers, and blood glucose monitors are essential [22]. This includes continuous blood pressure monitoring for patients with hypertension, smart inhalers for monitoring COPD, and real-time glucose tracking for managing diabetes [23].

b. Remote Patient Monitoring (RPM)—Heart rate, blood pressure, temperature, and other health data can be automatically collected through wearables and portable ECG monitors and sent to a software program where algorithms can evaluate the information to recommend treatments or generate alarms [24]. RPM makes it easier to track health in real time, which improves care for older people who live independently and enables preventive treatments for high-risk patients [25]. Healthcare professionals may monitor patient health in real time thanks to the continuous flow of data, which reduces the need for frequent in-person visits.

c. Preventive Healthcare—Fitness trackers and smartwatches aid in health maintenance by monitoring physical activity, sleep patterns, and stress levels through heart rate variability analysis [26]. These devices promote proactive well-being management and healthier lifestyle choices.

#### 2.1.2. Relevance to Connected Ecosystems

IoT-enabled solutions facilitate smooth integration and real-time interaction between patients, healthcare providers, and medical devices across many healthcare stakeholders, laying the groundwork for connected health ecosystems [27].

Critical health and sensitive patient data are continuously gathered by a variety of medical devices and sent via the Internet for further analysis and decision making in an interconnected environment made possible by IoT technologies [28,29]. Real-time interactions are made possible through the seamless communication between these systems and devices inside the ecosystem, which also keeps patients, physicians, hospitals, and caregivers informed. Through the aggregation of data from several IoT devices, this integration promotes a linked healthcare network and offers comprehensive insights.

Cloud-based platforms and data integration technologies are used by connected health ecosystems to facilitate the gathering, secure storage, and analysis of patient data from IoT devices [30], improving decision making and enhancing collaboration across healthcare institutions.

Through the continuous analysis of patient data within the ecosystem and the provision of real-time health insights by means of wearables or applications, IoT-enabled monitoring enhances proactive and personalized care [31]. Furthermore, healthcare practitioners receive early warning alerts from AI-driven analytics, which helps avoid complications and decrease the number of hospitalizations.

Regardless of geographical location, connected ecosystems in healthcare bring together different stakeholders—hospitals, clinics, patients, and devices—into a cohesive framework that improves collaboration and provides treatment continuity [32]. For enhanced overall efficiency, hospitals deploy IoT systems for equipment optimization, resource management, and patient tracking.

These ecosystems also contribute to population health monitoring by detecting trends like disease outbreaks or high-risk patients [33], enabling targeted interventions through the utilization of data-driven insights [34].

Seniors can live independently with personalized care plans and remote monitoring thanks to the seamless integration of smart home health systems and linked wearables into connected ecosystems. Smart home health systems further integrate wearable devices, allowing seniors to maintain independent living with continuous health support [35].

### 2.2. Challenges in IoT-Based Health Monitoring

Despite IoT benefits for health monitoring, a number of challenges prevent its widespread adoption of IoT-enabled healthcare systems. These challenges span the technical, operational, and ethical dimensions [36]:

- *Data accuracy and reliability:* ensuring that IoT devices provide accurate and consistent health data for effective decision making;
- *Data security and privacy*: strong data encryption, authentication mechanisms, secure transmission in IoT health applications, and compliance with healthcare regulations like GDPR (EU) and HIPAA (USA) are required to protect sensitive patient information;
- *Infrastructure and connectivity:* reliable internet connections (such as Wi-Fi and 5G) and sufficient network capacity are essential for real-time data transmission;
- *Energy efficiency:* continuous data transmission in wearable devices must be optimized for low power consumption to extend battery life;
- *AI and machine learning integration*: implementing real-time anomaly detection and predictive analytics necessitates powerful computers and optimized software;
- *Interoperability and scalability:* the adoption of interoperability frameworks (such as FHIR) is necessary to ensure the seamless integration of various IoT devices and healthcare systems;
- *Ethical considerations*: addressing issues with patient autonomy, consent, and data ownership is critical for responsible IoT implementation in healthcare;
- *Cost and accessibility:* removing financial obstacles for smaller healthcare providers and guaranteeing fair access to IoT-enabled healthcare, especially in low-income or vulnerable regions, are important for avoiding a digital divide.

Resolving these issues is essential to guaranteeing the success of connected health ecosystems and requires continuous innovation and cooperation between producers, healthcare providers, and legislators.

### 2.3. Communication Technologies for IoT Healthcare

Efficient communication protocols are fundamental to IoT-driven healthcare systems, ensuring real-time monitoring, secure data transmission, and minimal latency for many IoT-driven healthcare applications in order to guarantee real-time monitoring and timely alerts. For example, rapid data transmission is necessary for emergency response apps, smart wearables, and remote patient monitoring systems to identify critical medical problems like hypoglycemia or cardiac arrhythmias.

#### 2.3.1. Latency and Bandwidth Challenges

Significant difficulties arise from latency and bandwidth constraints, especially in settings with a high device density or unreliable network infrastructure. Many IoT healthcare applications require ultra-low latency to ensure timely alerts and immediate responses in many IoT health applications, like insulin administration systems, fall detection, and cardiac monitoring, to guarantee prompt reactions in emergency situations. The effectiveness of treatment and patient safety could be compromised by any delay in data transmission.

These issues are addressed by several wireless technologies with differing trade-offs:

- Wi-Fi’s fast transmission rates make it ideal for sending massive amounts of medical data, including continuous vital sign monitoring or high-resolution imaging. It can be used for high-bandwidth medical image transfers and real-time video consultations, but its high-power consumption limits it from being used in battery-operated devices [37].
- BLE is intended for low-power consumption, making it perfect for wearable health devices and short-range communication between medical sensors and smartphones, yet its short range prevents widespread coverage in hospital settings or remote monitoring scenarios [38].
- LoRaWAN offers long-range connectivity with low power consumption, which makes it ideal for asset tracking in hospitals and rural healthcare applications for sending small health-related data packets over long distances. However, because of its low data rate, LoRaWAN is not appropriate for sending high-resolution medical data [39].

Communication bottlenecks in high-density environments or unreliable network infrastructure can hinder real-time patient monitoring. In order to maximize effectiveness, reliability, and real-time responsiveness in IoT-driven healthcare systems, a hybrid strategy combining many wireless technologies is needed to address these latency and bandwidth limitations.

#### 2.3.2. Energy Constraints and the Need for Low-Power IoT Communication

With the use of wearable and implanted technology, remote health monitoring systems are essential to contemporary healthcare since they allow for continuous patient surveillance. Managing energy restrictions to increase device longevity and guarantee dependable performance is an essential challenge in these systems.

Power-Efficient Sensing and Transmission: Wireless Body Area Networks, which are made up of sensor nodes that run on batteries and gather and send physiological data, are frequently utilized in health monitoring. Since regular battery replacements are impractical, particularly for implantable devices, energy efficiency in these networks is essential. Research indicates that the transmission and processing of data uses a lot of energy. Low-power communication protocols such as BLE and LoRaWAN offer extended battery life while ensuring reliable data transmission.

Techniques that include eliminating redundant data at the sensor level, lowering the amount of transmitted data, and conserving energy have been suggested as ways for reducing this [40].

#### 2.3.3. Advances in Low-Power Communication Protocols

The primary objective of communication protocol advancements has been to utilize less energy. Emerging protocols such as NB-IoT and Zigbee optimize data transmission efficiency while minimizing power consumption. Low-power communication protocols such as BLE and LoRaWAN offer extended battery life while ensuring reliable data transmission. The treNch protocol is capable of lowering power consumption by one to two orders of magnitude in comparison to various BLE modes in a variety of scenarios while maintaining an equivalent level of service quality [38,41].

#### 2.3.4. Energy Harvesting Techniques

One possible solution to address energy limits is to incorporate energy harvesting techniques into IoT devices. Battery power can be substituted or supplemented by methods including solar, thermal, and kinetic energy harvesting, allowing devices to run for extended periods of time between charges [42,43,44,45].

A variety of strategies, such as energy harvesting methods, sophisticated low-power communication protocols, and power-efficient sensing, are used to address energy limits in remote health monitoring.

#### 2.3.5. Cloud vs. Edge Computing in Healthcare IoT

In healthcare IoT systems, the choice between cloud and edge computing architectures significantly impacts performance, particularly concerning latency and bandwidth [46,47].

*Cloud-Based IoT Platforms*: Cloud computing has significant scalability. However, because data must be transmitted from the IoT devices to the cloud and back, this centralized solution may cause latency problems and network dependency. Even though cloud computing enables scalability, in time-sensitive healthcare applications where instantaneous data processing is essential, such delays could be critical [48].

*Edge Computing Solutions*: By processing data locally on embedded devices or nearby servers, edge computing overcomes these issues by lowering latency and bandwidth consumption. Real-time data analysis and decision making are made possible through this decentralized method, which is essential for applications like emergency response and patient monitoring [49].

### 2.4. Sensor Fusion and Data Processing in IoT Health Systems

In order to produce a more accurate and complete representation of a subject or environment, multi-sensor fusion enhances the accuracy and reliability of health monitoring by combining data from multiple sources such as motion sensors, biomedical sensors, and environmental sensors, producing insights faster and allowing for more sophisticated analysis.

#### 2.4.1. Importance of Sensor Fusion for Accurate Health Monitoring

Sensor fusion enables a more comprehensive understanding of patient health, enabling activity monitoring through the integration [50] of multiple sensor modalities. For example, combining electrocardiography (ECG) with motion-tracking technologies enhances cardiac monitoring accuracy.

Some examples of multi-sensor fusion applications are provided below:

*Gait Analysis*: Combining wearable technology with data from vision-based systems, such as Kinect sensors, improves the comprehension of joint movements in gait analysis and results in more accurate evaluations of gait anomalies [51,52].

*Vital Signs Monitoring*: By combining information from many biomedical sensors, vital signs can be monitored more precisely, which enhances the identification of disorders including arrhythmias and sleep apnea [53,54].

*Activity Recognition*: Through activity tracking, multimodal fusion sensors in body-worn devices have enhanced physical activity monitoring, preserving or improving the quality of social and personal lives [55].

#### 2.4.2. AI and Machine Learning for Predictive Analytics

Advanced AI-driven analytics improve healthcare decision making by detecting health anomalies, predicting disease progression, and offering personalized treatment recommendations.

#### 2.4.3. Emerging Trends in Multi-Sensor Data Processing

Recent advancements in wearable technology and edge computing facilitate real-time data analysis while minimizing network dependency. AI-enhanced data filtering techniques enhance data accuracy and decrease redundant transmissions.

These advancements in sensor fusion technologies are essential for facilitating medical diagnosis and improving healthcare quality.

This literature review has examined key advances, challenges, and emerging trends in IoT-enabled healthcare monitoring. By leveraging multi-sensor fusion, AI-driven analytics, and efficient communication protocols, IoT-based health ecosystems have the potential to enhance real-time patient monitoring and personalized healthcare delivery. However, addressing challenges related to data security, interoperability, and energy efficiency remains critical for the widespread adoption of IoT in healthcare.

## 3. Materials and Methods

### 3.1. Monit4Healthy System Architecture and Design

**Figure 1:**
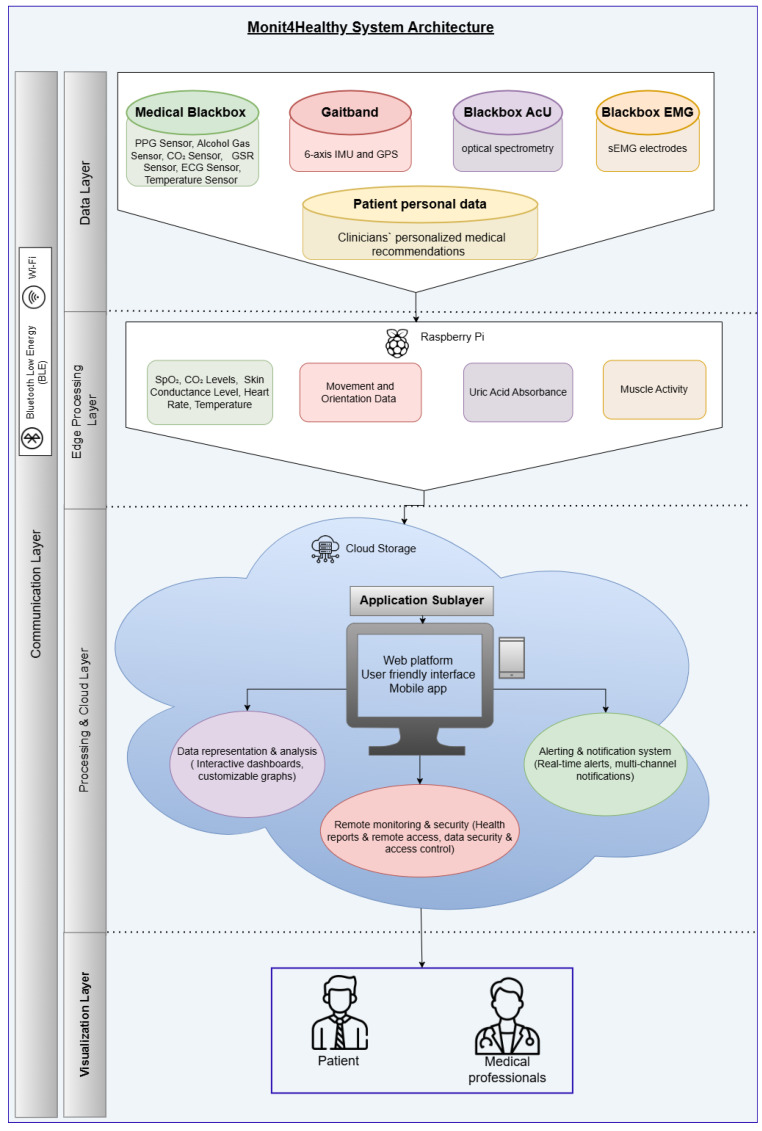
Overview of the Monit4Healthy system architecture, showing the interaction between data collection (wearable and portable medical devices), wireless communication (BLE and Wi-Fi modules), edge processing (Raspberry Pi), cloud storage and analytics (ICIPRO platform), and visualization layers (clinician dashboards and mobile interfaces). The diagram highlights the structured flow of biomedical data—acquired via multi-sensor fusion—transmitted securely from the Medical Blackbox and other devices to the cloud infrastructure for real-time monitoring, anomaly detection, and visualization within connected health ecosystems.

The Monit4Healthy system was designed using a modular sensor fusion architecture that ensures scalability, interoperability, and real-time data acquisition. The system is structured across multiple levels. The architecture consists of five core layers:

- *Data Layer*: This layer involves the real-time collection of physiological and environmental data using advanced biomedical sensors integrated into multiple monitoring devices.
- *Communication Layer*: This layer facilitates efficient and secure data transmission. Wearable devices rely on BLE for short-range communication, while high-bandwidth data transfers occur via Wi-Fi to ensure seamless connectivity with cloud-based storage and processing systems.
- *Edge Processing Layer*: This layer is responsible for local data processing and initial analysis before transmitting physiological and biomechanical data to the cloud. This layer operates on a Raspberry Pi, which processes raw sensor signals from the Medical Blackbox, AcU Blackbox, EMG Blackbox, and Gaitband IoT-based devices (designed and developed for the Monit4Healthy system). Compared to a cloud-only setup, this approach improves latency, minimizes bandwidth usage by preprocessing data locally, and ensures real-time responsiveness. The implementation of edge computing in IoT systems introduces several significant advantages: (1) reduced latency, by processing data directly at the edge nodes, ensuring fast response times essential for real-time healthcare monitoring; (2) bandwidth efficiency, as only relevant, processed data are transmitted to cloud servers, reducing network congestion; (3) system autonomy and fault tolerance, allowing continued local operation even when cloud services are temporarily unavailable; and (4) enhanced privacy and security, as sensitive patient data are filtered and processed locally, limiting external exposure and improving compliance with data protection standards. Figure 1 highlights the data flow between edge and cloud layers [56].
- *Processing and Cloud Layer and Application Sublayer*: The Processing and Cloud Layer is responsible for data storage, structured representation, and alerting mechanisms. This layer aggregates sensor data transmitted from the Edge Processing Layer, organizing them for structured analysis and long-term storage. It includes interactive data processing tools, enabling customizable health metric representations and structured reports. A cloud-based alert and notification system operates within this layer, continuously analyzing incoming physiological and biomechanical data. It detects deviations from predefined health thresholds and triggers automated alerts through multi-channel notifications. Secure storage mechanisms are integrated to ensure data encryption, controlled access, and regulatory compliance for sensitive health information. The Application Sublayer acts as the central processing interface for managing sensor data, health alerts, and system configurations. This layer facilitates remote monitoring functionalities, allowing healthcare providers to manage real-time and historical patient data. It is responsible for user authentication, system configuration, and data synchronization across cloud servers and edge devices. It also handles communication between medical professionals and patients, ensuring the seamless integration of monitoring data with personalized medical recommendations.
- *Visualization Layer*: This layer focuses on presenting real-time health insights in a user-friendly format. It provides interactive interfaces for different stakeholders, including patients, clinicians, and researchers, ensuring efficient access to health data. Features include dynamic dashboards, trend graphs, and anomaly detection alerts, ensuring that medical professionals and patients receive timely information.

To guarantee organized aggregation as well as secure handling, real-time health data gathered from wearable devices are sequentially processed through many levels in the Monit4Healthy system. In addition to transmitting raw sensor data, the shift from edge computing to cloud infrastructure additionally involves preprocessed signals that have already been filtered and structured for additional analysis. The integrity and accuracy of physiological and biomechanical parameters are maintained while bandwidth consumption is reduced using this sequential method.

The structural organization and interaction between these layers are visually represented in Figure 1, which illustrates the Monit4Healthy system architecture. This figure provides a comprehensive overview of how data flow from the initial sensor measurements to real-time decision-making and visualization tools. It highlights the integration of hardware and software components, showcasing the seamless connectivity between IoT devices, cloud infrastructure, and end-user applications. This architecture was designed for ensuring the system’s ability to deliver accurate, efficient, and scalable health monitoring solutions.

The Monit4Healthy system integrates a suite of specialized biomedical IoT-based devices, each designed for specific health monitoring applications:

- *Medical Blackbox*: A multifunctional enclosure that integrates various medical-grade sensors, designed to facilitate efficient data acquisition and processing. It captures and analyzes vital parameters such as heart rate, SpO_2_, carbon dioxide levels, and GSR. This device integrates high-precision sensors for real-time physiological assessment, ensuring the accurate detection of health anomalies.
- *Gaitband*: A wearable device designed for real-time gait pattern monitoring, optimized for ergonomic comfort and sensor placement stability. Equipped with an LSM303DLHC magnetometer and accelerometer, the Gaitband continuously monitors gait patterns, posture stability, and movement dynamics. The SAM-M8Q GPS (Global Positioning System) module enhances location tracking and movement analysis, while the HM-10 Bluetooth module and ESP8266 (NodeMCU) facilitate secure wireless data transmission. By analyzing motion data, the device detects irregular walking patterns, potential fall risks, and neuromuscular impairments, providing valuable insights for mobility assessment and rehabilitation.
- *EMG Blackbox*: A specialized enclosure engineered for housing the EMG sensor array, ensuring both signal integrity and protection from external interference. Utilizes surface electromyography (sEMG) sensors for neuromuscular function assessment, measuring electrical muscle activity to monitor conditions such as muscle fatigue and neurodegenerative disorders.
- *AcU Blackbox*: A device that employs advanced optical spectrometry techniques for non-invasive uric acid monitoring in urine samples. The device provides real-time biochemical analysis, enabling the early detection of metabolic imbalances.
- *Patient personal data*: Based on personal medical history and previous measured parameters and information from the Monit4Healthy database, clinicians can issue personalized medical recommendations to the patients under their care, in order to communicate clinical or lifestyle actions that the patient should follow. Each recommendation comprises several attributes, such as the title, an optional reference to the medical device that the patient might use in order to complete the action, and the medical recommendation from the physician. The title serves as a brief summary of the action, while the description offers details and guidance for the patient. The medical device is optional, mentioned if the patient needs to use it in order to collect data for ongoing assessment or monitoring, e.g., the Gaitband, for movement analysis. It is a means of communication between clinicians and patients, allowing physicians to remind patients of the necessary clinical or lifestyle actions they should undertake to improve their health. These actions may include tasks such as performing daily exercise, using a medical device to acquire specific data, or scheduling appointments with medical specialists. Each medical recommendation is uniquely associated with the medical professional and their assigned patient, and it is time-stamped and can be easily tracked. The recommendation’s title, associated device, and description are all stored with structured data fields to allow easy retrieval and management.

Each device transmits data securely via the Monit4Healthy gateway (Raspberry Pi), which preprocesses and optimizes data for cloud storage.

### 3.2. Blackbox Devices’ Concept and Development

The Blackbox devices within the Monit4Healthy system follow a modular sensor-fusion framework, integrating low-power microcontrollers (ESP32, STM32) for energy-efficient processing. Edge computing optimizes local data analysis, reducing bandwidth usage and improving response times. To ensure data security, encryption protocols and secure communication channels are implemented, aligning with regulatory standards.

For precise integration and manufacturability, Autodesk Fusion 360 [57,58] was chosen due to its all-in-one 3D modeling, parametric design, simulation, and real-time collaboration capabilities. This cloud-based platform enabled efficient transitions from concept to production.

Development began with manual measurements and hand sketches, later digitized in Autodesk Fusion 360 to ensure accuracy in geometric constraints, tolerances, and material properties. This structured workflow, applied to all Blackbox devices, minimized design inconsistencies and optimized fabrication processes.

The design process included defining functional specifications, developing 2D sketches, and transitioning to parametric 3D models with manufacturability optimizations (e.g., fileting, draft angles, sensor housings). High-fidelity simulations (FEA, thermal analysis) ensured structural robustness and hardware integration. Iterative refinements addressed discrepancies, optimizing material use and production feasibility.

Final designs were formalized in STL format for seamless fabrication, ensuring scalability and real-world deployment. The combination of manual sketching, digital modeling, and iterative validation enhanced reliability and performance, as illustrated in subsequent figures.

**Figure 2:**
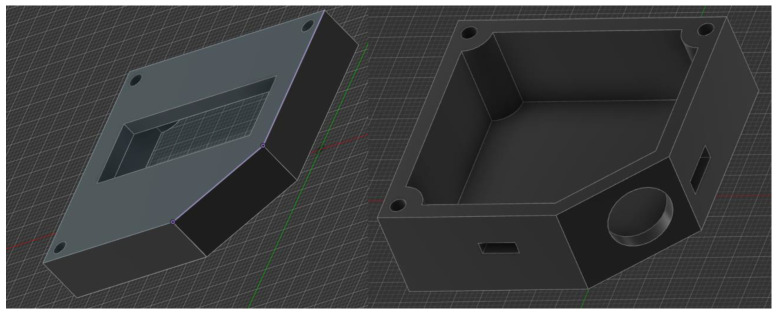
The final model of the EMG Blackbox. The final assembled prototype of the EMG Blackbox, showcasing its compact design for portable biomedical monitoring applications. The image highlights key components, including the embedded microcontroller board (Arduino Mega), Li-Po battery pack (7.4 V, 2500 mAh), wireless communication module (NodeMCU ESP8266), and integrated EMG signal acquisition sensors. Internal wiring and modular connectors are organized to support reliable signal acquisition and efficient power distribution. This configuration allows for real-time data processing and wireless transmission to edge computing nodes, ensuring robust operation in laboratory and home-care settings.

The finalized prototype of the EMG Blackbox is illustrated in Figure 2, showcasing its compact design, component layout, and integration of core hardware elements such as the Arduino Mega, NodeMCU ESP8266, and Li-Po battery. This configuration supports real-time data acquisition, wireless transmission, and portable biomedical monitoring capabilities.

Figure 2 illustrates the 3D extruded model derived from a 2D sketch in Autodesk Fusion 360. The enclosure is designed for housing electronic or medical sensors and features:

- Corner mounting holes for secure fastening.
- Side cutout ventilation and USB-C connectivity.
- A circular opening on the angled face, for sensor integration.
- *Geometric features*: Rectangular base with an inclined edge for structural compatibility; the chamfered surface enhances ergonomics and esthetics.
- *Mounting points*: Four precisely positioned screw holes ensure secure fastening and stability.
- *Recessed cavity*: Central section accommodates a display, sensor, or functional interface.
- *Manufacturing considerations*: Optimized for 3D printing, balancing material efficiency and structural integrity.

**Figure 3:**
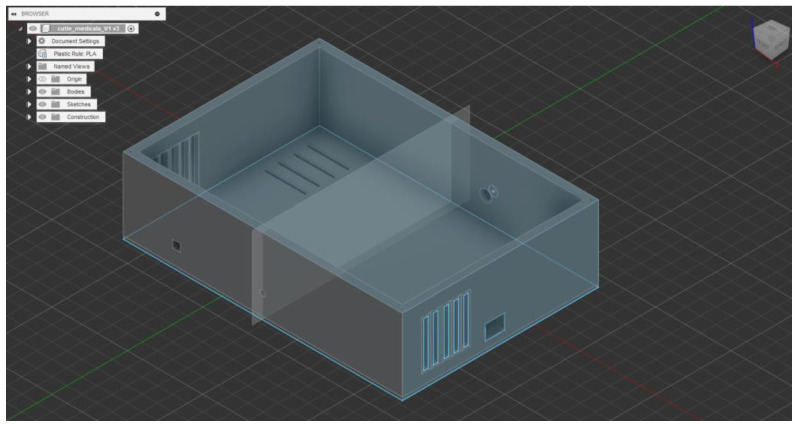
The final model of the Medical Blackbox. The finalized prototype of the Medical Blackbox device used in the Monit4Healthy system. The image shows the internal layout of key components, including the Arduino Mega microcontroller, signal conditioning circuits, NodeMCU ESP8266 for wireless communication, and the Li-Po battery pack. The device integrates multiple biomedical sensors (ECG, EMG, photoplethysmography (PPG), GSR) for real-time physiological data acquisition and processing. The compact and modular design supports portable operation in clinical and home environments, facilitating edge-level signal processing and secure data transmission to cloud infrastructure.

Figure 3 illustrates the fully developed 3D model of the Medical Blackbox, designed in Autodesk Fusion 360 for the Monit4Healthy system. The enclosure integrates structural, functional, and ergonomic optimizations for housing medical-grade electronics and sensors.

*Technical and functional design considerations*

- *Structural optimization*: Rigid, lightweight construction ensuring mechanical protection and spatial efficiency.
- *Ventilation system*: Engineered side slots for effective heat dissipation and sensor accuracy.
- *Custom interface openings*: Strategically placed cutouts for sensors, power, and data ports, minimizing interference.
- *Fastening and accessibility*: Predefined mounting holes and alignment guides facilitate secure assembly and maintenance.
- *Manufacturing readiness*: Optimized for 3D printing, ensuring precision, repeatability, and material efficiency.

*Key design considerations*

- *Dimensional accuracy:* Defined length/width parameters for precise fit and structural integrity.
- *Feature placement:* Central cutout for connectors, sensors, or ventilation.
- *Alignment and constraints:* Geometric symmetry ensures parametric adaptability.
- *Manufacturing preparation:* Sketch optimized for extrusion, fileting, and fastening integration.

**Figure 4:**
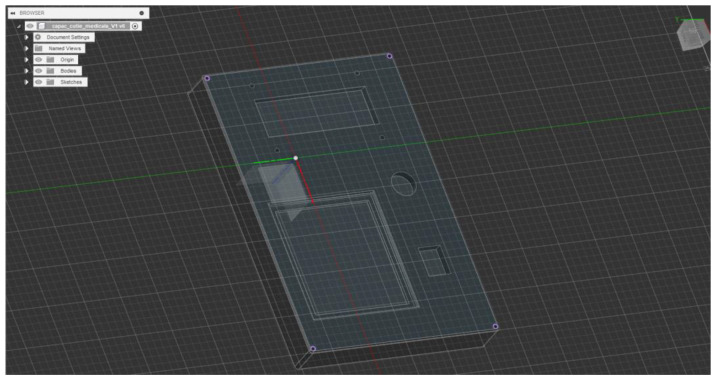
The finished model of the Medical Blackbox’s lid. The finalized model of the Medical Blackbox’s external casing, specifically its custom-designed lid. The enclosure was designed to ensure physical protection for internal electronic components, including the microcontroller, sensors, and communication modules, while maintaining accessibility for maintenance and upgrades. The lid incorporates ventilation slots for thermal management and designated ports for sensor connections, power input, and wireless communication interfaces, ensuring reliable and safe operation during portable health monitoring activities.

Figure 4 presents the fully developed 3D model of the Medical Blackbox cover, designed for seamless integration with the enclosure. The model incorporates precisely positioned cutouts, fastening points, and structural reinforcements to enhance functionality and system performance.

- *Component-specific cutouts*: Geometrically optimized openings for sensor exposure, display integration, and thermal dissipation.
- *Precision fastening*: Predefined corner mounting holes ensure secure, vibration-resistant attachment while maintaining accessibility.
- *Multi-plane design*: Slanted and recessed surfaces enhance ergonomics, esthetics, and mechanical robustness.
- *Manufacturing and assembly readiness*: Optimized for additive/subtractive manufacturing, ensuring tight tolerance and reliable interlocking with system components.

*Data Transmission Framework*

Through the use of BLE and Wi-Fi protocols, which both enable Transport Layer Security (TLS) 1.2/1.3 and Secure Socket Layer (SSL) for encrypted communication, ensuring authentication and integrity, the Monit4Healthy system enables secure information exchange. TLS guarantees all external data transmissions to cloud services, whereas BLE is employed for local, short-range biomedical data communication. In particular, the system uploads data to the ICIPRO cloud infrastructure—which is designed for the secure processing and storage of sensitive data—using TLS-encrypted communication. The ICIPRO infrastructure provides certified secure settings for digital health solutions. As part of its compliance to national cybersecurity regulations, ICIPRO uses TLS protocols to establish server-side encryption and enforces data isolation, access control constraints, and encrypted backups. The system uses an adaptive data packet framework, resulting in only pertinent and priority information for transmission, edge-level data preprocessing, and this lowers the total amount of raw signals, and dynamic protocol selection depending on the operational context to accomplish this.

In particular, Wi-Fi transmission facilitates the transfer of higher-resolution biomedical data to cloud platforms for additional analysis, whilst BLE allows for short-range, low-power communication among sensors and local processing units. This hybrid communication approach ensures low-latency data sharing, which is important for real-time health monitoring, while optimizing energy efficiency and network bandwidth consumption. The system constantly determines between BLE and Wi-Fi in order to improve data transmission by considering the trade-offs between data throughput, communication range, and power efficiency. Although BLE guarantees minimal power consumption for short-range exchanges, its data speeds are constrained, and it performs most effectively for sending low-volume, sensitive information. The reliable transmission of high-resolution biomedical data is made possible by Wi-Fi’s greater bandwidth and range, but at the expense of higher power consumption.

The intrinsic interference mitigation approaches included into BLE and Wi-Fi protocols are used to lessen any signal interference in high-density environments. To further boost transmission accuracy under overloaded network contexts, future system versions might consider adaptive communication approaches.

The following sections present the key modules and protocols utilized within the system, emphasizing their role in enabling seamless and secure data exchange.

*Data Flow Structure Within the Monit4Healthy System*

Various sensors track vital parameters such as heart rate, SpO_2_, temperature, and EMG signals. These sensors communicate through standardized wireless protocols, ensuring seamless data transmission.

The edge level of the Monit4Healthy system is where integrated feature extraction and multi-sensor data synchronization are carried out. Individual preprocessing stages (such as noise filtering, timestamp alignment, and feature extraction) are applied to physiological signals from ECG, EMG, PPG, and GSR sensors. The features extracted are subsequently categorized into integrated data packets for transmission and real-time analysis. At the microcontroller level, each signal is subjected to distinct filtering and amplification based on its frequency and amplitude features. This is complemented by analog-to-digital conversion and timestamp-based alignment. After being synchronized, the signals are sent to an edge computing unit for initial signal processing. Despite the current lack of a formal fusion approach (such as rule-based fusion), the system architecture enhances context awareness by supporting simultaneous data processing and displaying correlated physiological parameters in real time. Future stages of development will involve the integration of advanced sensor fusion algorithms to provide complex health metrics based on multi-sensor inputs.

**Figure 5:**
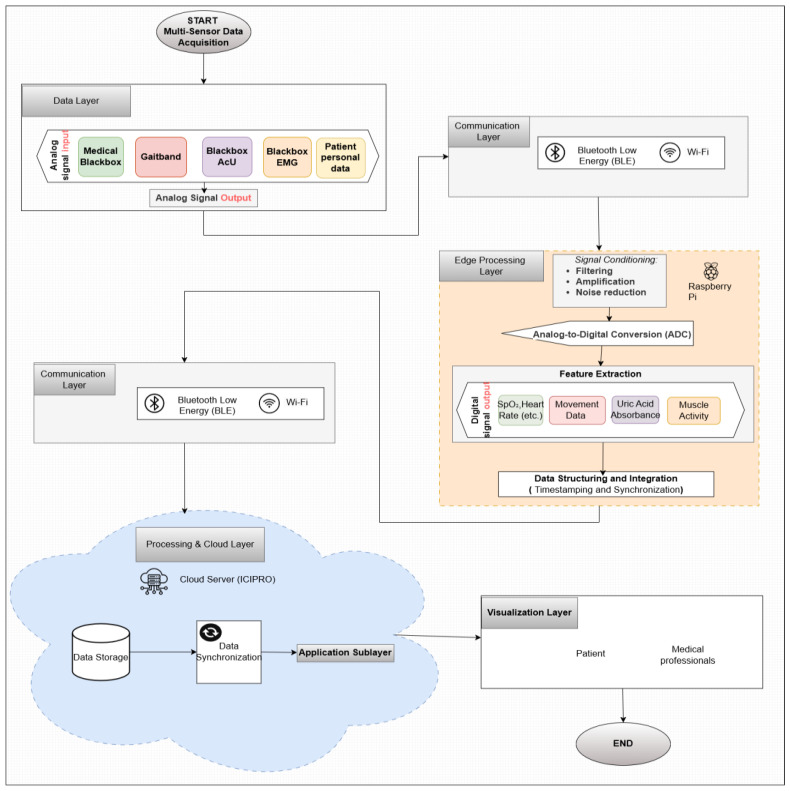
Multi-sensor data acquisition, synchronization, and structured transmission workflow in the Monit4Healthy system.

The data flowchart in Figure 5 illustrates the process of multi-sensor data acquisition, synchronization, preprocessing, and transmission in the Monit4Healthy system. It represents the flow of data from initial acquisition through multi-sensor systems to final data transmission, ensuring that the physiological signals from the patient are accurately monitored, processed, and transmitted to healthcare professionals for further analysis and timely interventions. A detailed description of the data flow and processing workflow is presented as follows:

- *Data Layer—Multi-Sensor Data Acquisition:* The process starts with the Data Layer, where physiological signals are continuously collected by various sensors integrated within the Monit4Healthy system. The key sensors involved in data acquisition include the Medical Blackbox, Gaitband, Blackbox AcU, and EMG Blackbox.
- *Communication Layer—Data Transmission:* Once the data are collected by the sensors, they are transmitted through two communication channels:
  ○ BLE, used for short-range communication, ideal for wearable or portable sensors that require low-power consumption.
  ○ Wi-Fi, used for higher-bandwidth, long-range data transmission, enabling the system to send large datasets such as high-resolution ECG and other complex signals to the cloud for further processing.
- *Edge Processing Layer—Initial Signal Processing:* After the data are transmitted, they enter the Edge Processing Layer. Here, the raw signals undergo initial processing, including the following:
  ○ Signal Conditioning: The filtering and amplification of the data to remove noise and enhance signal quality.
  ○ Feature Extraction: Relevant features such as heart rate from ECG and PPG signals, muscle activity from EMG, and skin conductance levels from GSR are extracted for further analysis.
  ○ Analog-to-Digital Conversion: The analog signals from the sensors are converted to digital format to facilitate further computational processing.
  A Raspberry Pi or similar edge computing devices are used in this layer to perform the initial processing, reducing the amount of raw data sent to the cloud and ensuring that only essential information is transmitted, thus minimizing bandwidth usage and latency.
- *Data Structuring and Integration:* Once the feature extraction is performed, the processed data are structured and integrated for synchronization across multiple sensors. This step ensures that the data from all sensors are aligned in time by applying timestamps, facilitating multi-sensor data fusion and providing a more comprehensive evaluation of physiological conditions. The integration of data from various sensors such as ECG, EMG, and GSR enhances the system’s ability to detect abnormalities and generate alerts when necessary.
- *Data Transmission to the Cloud:* After data fusion, the structured data are transmitted using the communication protocols (BLE or Wi-Fi) to the cloud server (ICIPRO). This enables the following:
  ○ Data Storage: The cloud serves as the repository for the processed data, ensuring they are stored securely and accessible for further analysis.
  ○ Data Synchronization: The real-time synchronization of the patient’s health data across devices and systems allows healthcare professionals to access the most up-to-date information at any time.
- *Application Sublayer—Real-Time Monitoring and Alerts:* At this stage, the structured and synchronized data are accessible via an Application Sublayer, where healthcare professionals and patients can view the data in real time through interactive dashboards. These dashboards provide visualizations such as the following:
  ○ Trend graphs showing the patient’s health metrics over time.
  ○ Alerts generated when the data deviate from predefined thresholds, indicating potential health risks. This allows for timely interventions, as healthcare providers can receive immediate alerts if a patient’s vital signs indicate a need for attention.
- *Visualization Layer—Health Data Visualization:* Finally, the processed data are displayed in the Visualization Layer, providing clear and easy-to-interpret health metrics for both patients and medical professionals. This layer facilitates the following:
  ○ The real-time visualization of key health parameters such as heart rate, SpO_2_, gait patterns, and muscle activity.
  ○ Alert notifications that inform healthcare providers about critical changes in the patient’s condition.
  ○ Access to multiple functionalities that provide real-time, updated data and information related to the patient’s profile, based on the information stored in the system’s database. These functionalities are considered and utilized as part of a personalized, proactive, and preventive health management support system for the patient.

*Communication Protocols and Optimization Strategies*

Efficient data exchange is crucial for real-time health monitoring systems. Wi-Fi and Bluetooth serve as the primary communication protocols for data transmission in IoT-enabled medical devices. Wi-Fi provides high-speed, long-range connectivity, enabling continuous data synchronization with cloud storage. The Bluetooth module ensures real-time data transmission by establishing a seamless wireless connection, which is vital for telemetry and remote monitoring applications.

- *Bluetooth Module for Real-Time Data Transmission:* The Bluetooth module facilitates real-time data transmission, ensuring reliable connectivity. This feature is essential for applications requiring continuous data flow, such as telemetry and remote patient monitoring. Advanced BLE technology ensures low power consumption and reduced latency, making it suitable for wearable health applications. It employs adaptive frequency hopping (AFH) to minimize interference and optimize bandwidth utilization. BLE and Wi-Fi are widely adopted wireless communication technologies for data transmission, each optimized for different application scenarios. BLE is designed for low-power, short-range communication, making it ideal for battery-operated devices such as wearable sensors and IoT nodes. In contrast, Wi-Fi provides higher data throughput and extended coverage, supporting applications that require continuous, high-bandwidth connectivity. Compared to other protocols such as Zigbee and LoRaWan, BLE offers higher data rates but at the cost of a reduced range, while Wi-Fi consumes significantly more power but ensures robust data transmission over greater distances. Although this work does not explicitly analyze the trade-offs in terms of power efficiency, range, and data throughput, it focuses on leveraging both BLE and Wi-Fi to optimize data transmission for specific use cases. BLE has significantly advanced from BLE4 to BLE5, delivering notable enhancements in communication reliability, the data transfer rate, and network scalability—essential elements for contemporary IoT and sensor network applications. Both BLE4 and BLE5 are designed primarily for low-power use—an important feature for battery-powered IoT devices and sensor networks. BLE4, launched with Bluetooth 4.0, utilizes a frequency-hopping spread spectrum technique within the 2.4 GHz ISM band to reach data rates of up to 1 Mbps, featuring a design tailored for energy efficiency and low-power functionality via simplified connection and advertising protocols. Conversely, BLE5 brings substantial improvements, including the adoption of a coded physical layer that boosts range and reliability in environments with high interference, along with a doubling of the nominal data rate to 2 Mbps. BLE5 increases the advertising capability by allowing larger payloads, which enhances detailed device discovery and connectionless communications. These technological advancements not only boost overall network efficiency but also allow for the incorporation of diverse sensors—like GPS units, EMG devices, and environmental sensors such as the DS18B20 and MAX30100—into more intricate and resilient monitoring frameworks. Both BLE and Wi-Fi are prone to interference in high-density environments. This is due to the shared spectrum in the 2.4 GHz ISM band, as well as due to the overlapping channels. To avoid congested channels, BLE utilizes adaptive frequency hopping (AFH). However, AFH’s effectiveness may diminish in dense environments, therefore leading to packet loss and reduced reliability. Wi-Fi networks experience co-channel interference when multiple access points operate on the same or adjacent channels, leading to slower data rates and increased latency, particularly in areas with many overlapping access points [59].
- *GPS Module:* The GPS module integrates a chip antenna and Qwiic cable connection, ensuring efficient hardware integration. It significantly reduces power consumption while enhancing performance and accuracy. The ability to receive signals from up to four Global Navigation Satellite System constellations simultaneously improves positional accuracy, particularly in environments with limited sky visibility, such as urban canyons or dense foliage. Additionally, Assisted-GPS technology is used to reduce the time-to-first-fix, ensuring faster positioning data. A differential GPS can also be employed to enhance precision, reducing errors to sub-meter accuracy. Based on the u-blox MAX-M10 GNSS platform, the GPS module used in the Monit4Healthy system supports simultaneous reception from up to four GNSS constellations (GPS, Galileo, GLONASS, and BeiDou), improving positioning accuracy and signal availability even in difficult-to-reach places like urban canyons. For power-optimized performance, it makes use of the u-blox Super-E technology, which uses as low as 25 mW while in continuous tracking mode. The Qwiic interface facilitates communication over I^2^C, allowing for effective data transmission with low latency and simplified wiring. In order to improve energy consumption in wearable health monitoring applications, the module incorporates automated low-power modes, such as power save and periodic tracking, and enables an Assisted Global Navigation Satellite System for an expedited time-to-first-fix. It has anti-jamming and anti-spoofing characteristics, which are essential for preserving data integrity in linked health ecosystems, and enables satellite-based augmentation systems for improved location precision. The MAX-M10 GPS module is a dependable and energy-efficient option for real-time geolocation in the Monit4Healthy system because of these features [60].
- *EMG Sensor (Myoware Sparkfun) for Muscle Signal Analysis:* This sensor measures EMG signals from muscle activity and features an integrated amplifier with an adjustable gain of up to 1000x. It includes a high-pass filter (0.5 Hz) to eliminate direct current offset and a low-pass filter (125 Hz) to reduce high-frequency noise. The sensor uses three disposable gel electrodes and outputs both raw and envelope signals, making it suitable for applications such as robotics and prosthetics. Signal conditioning is performed to reduce motion artifacts and interference from ambient electrical sources. A notch filter (50/60 Hz) is applied to mitigate power line interference. The Monit4Healthy system’s integrated EMG module uses a multi-stage analog signal conditioning architecture to record and process surface electromyography data with high fidelity. In order to separate the physiological EMG frequencies while reducing low-frequency motion artifacts and high-frequency noise, the signal collection chain starts with an instrumentation amplifier set up for an initial gain of 200× and operating within a passband of 20 Hz to 498 Hz. Signal scaling according to electrode location and specific muscle activation levels is made possible through the following gain stage, which may be adjusted between 1× and 100×. A 3.6 Hz high-pass filter is incorporated into the module to remove DC offset, guaranteeing baseline stability and reducing signal drift over time. After amplification, the signal passes through an envelope detection stage and a full-wave rectification circuit, enabling the recording of both rectified and envelope outputs. A high-resolution analog-to-digital converter digitizes these analog outputs, guaranteeing precise EMG signal sampling for edge processing. In the Monit4Healthy Edge Processing Layer, adaptive feature extraction and threshold-based event detection are made possible through the architecture’s support for the real-time monitoring of both raw and processed EMG data. With a low power consumption appropriate for wearable usage, its modular and adjustable architecture enables the EMG module to be tailored for a variety of health monitoring applications, such as neuromuscular evaluations and rehabilitation situations [61].
- *DS18B20 Digital Temperature Sensor:* The DS18B20 temperature sensor operates using a 1-wire interface, allowing multiple sensors to share a single data line. It provides accurate temperature readings with a configurable resolution (9–12 bits) and digital transmission to minimize errors compared to analog sensors, making it ideal for biomedical and environmental applications. The sensor includes an integrated temperature compensation feature, ensuring stable readings even under varying environmental conditions. It supports cyclic redundancy check for error detection, enhancing data integrity. The DS18B20 sensor was chosen for its networking advantages, using a 1-wire interface that allows multiple sensors to share a single data line, simplifying wiring in large networks. Data from the sensors were collected with a microcontroller and transmitted via a wireless connection to a cloud platform. The digital transmission of data reduces errors compared to analog sensors, providing accurate and reliable measurements over time [62].
- *MAX30100/MAX30102:* Pulse Oximetry and Heart Rate Sensors utilize photoplethysmography (PPG) to measure blood SpO_2_ and heart rate. In our project, the integration of the MAX30102 biosensor with the ESP32 microcontroller utilizes the Inter-Integrated Circuit (I^2^C) protocol to facilitate seamless data transfer, which is essential for monitoring key health parameters such as heart rate and SpO_2_ [63]. The sensor includes infrared and red light emitters for dual-wavelength analysis, improving accuracy by compensating for motion artifacts. An integrated ambient light cancelation system enhances signal clarity. The MAX30100 sensor measures PPG signals to determine the SpO_2_ levels and heart rate. Data are then sent wirelessly (via Bluetooth or Wi-Fi) to cloud storage, enabling real-time remote monitoring. This setup allows healthcare professionals to access patient data remotely and make timely interventions if necessary [64].

### 3.3. Development Environment and Tools

To ensure the seamless management of patient and medical professional data while maintaining secure accessibility, the Monit4Healthy system leverages robust web frameworks. The system integrates Django for backend processing, RESTful APIs for efficient data exchange, and secure authentication protocols for access control. Additionally, it supports medical professional management by handling medical results, tracking patient recommendation history, managing referrals, and overseeing the integration of patients with connected medical devices.

#### 3.3.1. Software Frameworks and Programming Environments

The Monit4Healthy system consists of a backend application developed using Python 3.13.1 and the Django 5.1.5 framework.

*Django Framework: A Secure Backend for Health Data Management*

Django, a high-level Python web framework, was selected for the Monit4Healthy system due to its advanced security model, scalable architecture, and native support for seamless interoperability with IoT ecosystems. Its built-in ORM, middleware capabilities, and modular design facilitate the development of a highly maintainable and performant backend, optimized for handling concurrent requests and large-scale data processing. These features are essential in the context of web-based and IoT-driven healthcare applications, where stringent data security, efficient real-time communication, and system resilience are critical requirements. Django’s architecture provides a robust and scalable framework ideally suited to the requirements of our project. A Django project encapsulates the entire web application, integrating all its components, while individual applications serve as modular units responsible for specific functionalities. The framework adheres to the Model–View–Template architectural pattern, ensuring a clear separation of concerns and promoting maintainability. The model layer defines the data schema and orchestrates database interactions, the view layer processes Hypertext Transfer Protocol requests and generates responses, and the template layer structures presentation logic, enabling the efficient rendering of response data in formats such as HTML or JSON. These characteristics make Django an optimal choice for developing a structured, maintainable, and high-performance web application [65].

Django’s Object Relational Mapper (ORM) provides an abstraction layer that simplifies database interactions by enabling developers to define and manipulate database models using Python code instead of raw SQL (Structured Query Language). This approach enhances code readability, maintainability, and portability while ensuring database-agnostic development, thereby facilitating seamless integration with various relational database management systems [66].

Django provides native support for a wide range of database management systems (DBMS), including MariaDB, PostgreSQL, MySQL, SQLite, and Oracle [67], through dedicated database drivers that handle connections and execute operations specific to each database management system.

For this system, the MariaDB management system was selected as the primary database management system due to its optimized performance, scalability, and extensive compatibility with Django’s ORM. It also enhances data storage and retrieval, ensuring fast and efficient access to patient records.

*Data Storage and Management: MariaDB*

This database provides the following:

- *SQL Support*: It facilitates the efficient and scalable querying, management, and manipulation of structured data.
- *Data Integrity and Security*: It implements strong encryption protocols and authentication mechanisms to protect sensitive health information, ensuring compliance with data protection regulations.

*The key functionalities implemented within the platform include the following:*

- *User Authentication and Role-Based Access Control*—Django’s built-in authentication system ensures that access to sensitive patient data is restricted to authorized personnel, enforcing strict role-based access policies.
- *Patient Management*—The framework enables the efficient storage and retrieval of patient data, appointment scheduling, and real-time notifications, ensuring seamless coordination between healthcare providers and patients.
- *Medical Professional Management*—Django supports the management of medical results, tracking of patient recommendation histories, and coordination of referrals. Additionally, it facilitates the integration of connected medical devices, with automated alert mechanisms notifying healthcare professionals of critical updates, such as abnormal test results or significant changes in a patient’s status.
- *Secure Data Handling*—The framework incorporates advanced security mechanisms, including built-in protection against SQL injection, cross-site scripting, and cross-site request forgery attacks, ensuring the integrity and confidentiality of medical data.

#### 3.3.2. Hardware Components Used

The Monit4Healthy system integrates a selection of high-performance microcontrollers and sensor technologies to enable real-time health monitoring and environmental data acquisition. These components ensure efficient data processing, seamless connectivity, and high reliability, which are critical for IoT-driven healthcare applications.

*Microcontrollers*

- *ESP32*—Chosen for its dual-core architecture, integrated Wi-Fi and Bluetooth capabilities, and ultra-low power consumption, the ESP32 is particularly suited for remote patient monitoring systems that require real-time data transmission over wireless networks. Its deep-sleep functionality minimizes energy usage, ensuring extended operation in battery-powered medical devices [68].
- *STM32*—These are a family of ARM Cortex-based microcontrollers offering high computational efficiency and low power consumption, making them ideal for embedded medical applications requiring real-time processing. An STM32 microcontroller compresses the digital ECG signals using a basic matrix–vector multiplication technique based on compressed sensing [69].
- *Raspberry Pi*—Serving as an edge computing node, Raspberry Pi offers high processing power and versatile I/O capabilities, enabling the real-time preprocessing of biomedical signals, anomaly detection, and encrypted data transmission to cloud servers. This reduces network latency and offloads computational tasks from the central cloud infrastructure [70].
- **ATmega2560 Microcontroller**—Applications requiring extensive firmware storage and multitasking can benefit from the ATmega2560, an 8-bit AVR microcontroller with 256 KB of flash memory, 8 KB of SRAM, and 4 KB of EEPROM. Four UARTs, an SPI, I^2^C, and sixteen 10-bit analog-to-digital converter channels are among the many peripherals it supports. The AT-mega2560, which runs at a clock speed of 16 MHz, provides dependable performance for activities involving real-time data collection and control while consuming little power, which is crucial for embedded health monitoring applications [71].
- **Arduino Mega Board**—With 54 digital input/output pins, 16 analog inputs, and various serial communication interfaces, the Arduino Mega board, which is based on the ATmega2560 microcontroller, has extensive I/O capabilities that enable integration with multiple biomedical sensors at once. The Arduino Mega is used in the Medical Blackbox device of the Monit4Healthy system to collect and interpret physiological signals in real time. Strong and scalable health monitoring systems are made possible by its dependable interrupt management, hardware timers, and interoperability with common sensor libraries, which provide effective and coordinated data collection from multi-sensor arrays [72]. Currently, the Medical Blackbox is powered via a regulated 5V DC input, compatible with external USB power adapters and battery packs, ensuring safe and portable operation during laboratory testing.
- *Biomedical Sensors*—Modules such as MAX30100, for heart rate and SpO_2_ monitoring via PPG; AD8232, for real-time ECG signal acquisition; a custom EMG module, for electromyography signal capture, designed with adjustable gain, bandpass filtering, and analog signal conditioning tailored for muscle activity monitoring; a GSR sensor, for galvanic skin response measurement, used in stress and autonomic nervous system assessments; and a digital temperature sensor, for continuous body temperature monitoring for real-time ECG signal acquisition, enable continuous physiological monitoring, facilitating the early detection of health anomalies.

*Hardware Integration and Connectivity*

In addition to the microcontrollers outlined above, the Monit4Healthy system incorporates a range of hardware components that support efficient sensor integration, signal acquisition, and wireless communication.

To support seamless multi-sensor integration and real-time data acquisition, the Monit4Healthy system utilizes modular hardware assemblies designed for efficient sensor interconnection and reliable data transmission. Each Blackbox device incorporates standardized connectors (e.g., Qwiic interface for I^2^C communication), allowing for rapid prototyping and scalability. Signal conditioning circuits, such as analog filters and amplifiers, are embedded within the hardware design to ensure high-fidelity signal acquisition and compatibility with microcontroller input ranges. Custom PCB layouts and compact enclosures, developed using parametric CAD tools, facilitate ergonomic deployment in clinical and remote settings. Wireless connectivity is achieved through integrated BLE and Wi-Fi modules, optimized for low-power consumption and robust communication, as detailed in Section 3.2.

#### 3.3.3. Data Processing Frameworks

*Edge and Fog Computing: Raspberry Pi as a Gateway*

The Monit4Healthy system employs edge computing paradigms to enhance data processing efficiency and reduce latency. Raspberry Pi serves as the central gateway, collecting sensor data and transmitting it to cloud infrastructure via Wi-Fi. The Raspberry Pi 4 Model B, equipped with wireless LAN and Bluetooth connectivity, plays a pivotal role in health monitoring applications by aggregating sensor readings, performing preliminary data processing, and ensuring real-time monitoring. This setup enhances system scalability, efficiency, and responsiveness, thereby improving both the accuracy and accessibility of patient data. With low power consumption (~2.5 W during typical operation) and optimized data transmission, the Raspberry Pi 4 Model B ensures energy-efficient performance, making it well suited for continuous health monitoring scenarios. Its capability to process and filter data locally before transmission significantly reduces network congestion and minimizes latency [73].

*Cloud Computing for Monit4Healthy System: ICIPRO IaaS*

ICIPRO—a significant cloud-based initiative designed to modernize and improve public services in Romania by leveraging cloud computing technology—provides Infrastructure as a Service (IaaS), offering virtualized computing resources, storage, and network connectivity through its secure cloud infrastructure. The platform is built on high-performance hardware, including servers, routers, and firewalls, ensuring reliability and scalability for systems like the Monit4Healthy system, which runs on a virtual machine (VM). ICIPRO provides high availability and load balancing, reducing downtime and improving system resiliency. VMs in this cloud run in a redundant data center environment, minimizing the risk of failure [74].

*Network Isolation and Security*

ICIPRO implements Network Virtualization using Generic Routing Encapsulation, ensuring complete network isolation for the Monit4Healthy system from other cloud tenants. A total of 10 Gbps of connectivity between hardware components provides low-latency and high-speed data transfers, essential for real-time health monitoring applications. Built-in firewall rules and virtual network segmentation enhance data security and prevent unauthorized access. ICIPRO integrates a System Center Data Protection Manager to offer continuous data backup and disaster recovery for critical health monitoring data [74].

**Figure 6:**
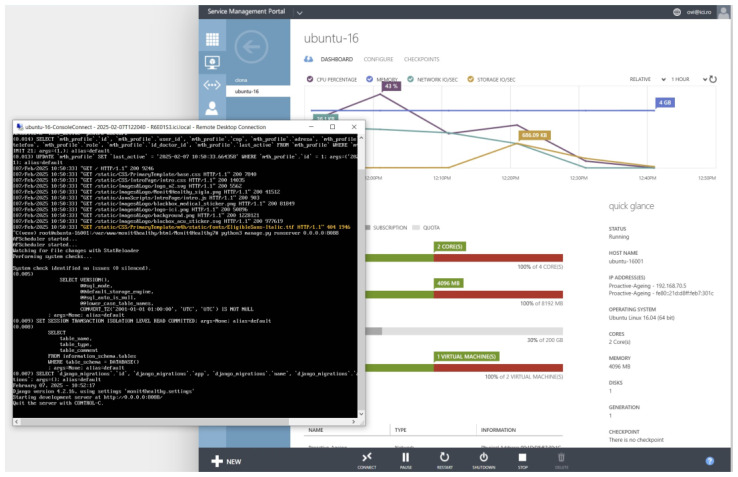
ICIPRO dashboard hosting the Monit4Healthy system’s cloud backend. The virtual machine runs Ubuntu OS with a Django development server (python3 manage.py runserver 0.0.0.0:8088), enabling secure data handling and real-time system monitoring through resource usage metrics and CLI interactions.

Figure 6 shows the ICIPRO dashboard controlling a virtual machine running Ubuntu, along with a terminal window—a Command Line Interface (CLI) running a Django development server using the “python3 manage.py runserver 0.0.0.0.0:8088” command—showing both system resource utilization and real-time CLIs. This architecture enhances predictive diagnostics and enables timely medical interventions, optimizing patient outcomes in a digitally connected healthcare ecosystem [75].

In addition to technical measures, data privacy and regulatory compliance are essential aspects of the Monit4Healthy system. The integration of cloud-based data transmission ensures low-latency, high-throughput, and encrypted data exchange, adhering to healthcare standards such as GDPR and, where applicable, HIPAA. Data privacy is maintained by implementing secure data transmission protocols, user authentication, and access control mechanisms within the system. The ICIPRO cloud infrastructure, where data are stored and processed, enforces strict security and privacy policies, including encrypted data transfer, controlled access, and secure storage, supporting compliance with GDPR and HIPAA principles related to health data confidentiality, integrity, and availability. In accordance with GDPR requirements, the system operates based on explicit informed consent from users, including healthcare professionals and patients, for data collection and processing. Consent procedures are designed to ensure transparency and user rights, including access, rectification, and data deletion upon request.

Using Python, HTML, CSS, PHP, SQL, and JavaScript for embedded programming, along with ICIPRO for UI and cloud integration, enables scalable and efficient software frameworks for real-time data exchange in connected health systems.

Throughout its sensor network, the Monit4Healthy system uses an efficient data processing framework that maximizes computing efficiency and minimizes energy usage. The technology preprocesses raw physiological signals locally before sending only relevant, organized data to the cloud through the use of edge computing at the device level. This approach ensures real-time sensitivity without using a large amount of power by effectively reducing bandwidth usage, network congestion, and cloud computing requirements. The Monit4Healthy system successfully establishes a balance between dependable data transfer and energy economy by integrating low-power transmission protocols like BLE for short-range communication. Through these procedures, the system optimizes total power consumption, preserves device battery life, and addresses key issues in latency-sensitive biomedical applications, therefore mitigating the typical limits of IoT health monitoring.

## 4. Results

The implementation of the Monit4Healthy system has advanced to the laboratory level, which includes the manufacturing of Blackbox devices and the associated monitoring platform, following the design and conceptual development phase. At this point, the system’s components—i.e., sensor-integrated Blackbox devices, data collection systems, early processing frameworks, and the platform—have been integrated and put through limited testing. Functional testing and validation, device calibration, and first performance evaluations were carried out in this phase, verifying that the basic architecture performs as planned prior to more extensive deployment considerations. The results, which demonstrate the system’s capabilities in a laboratory setting and its current degree of development, are shown in the following subsections.

### 4.1. Selected Illustrations of Implemented Devices

#### 4.1.1. The Medical Blackbox Device

**Figure 7:**
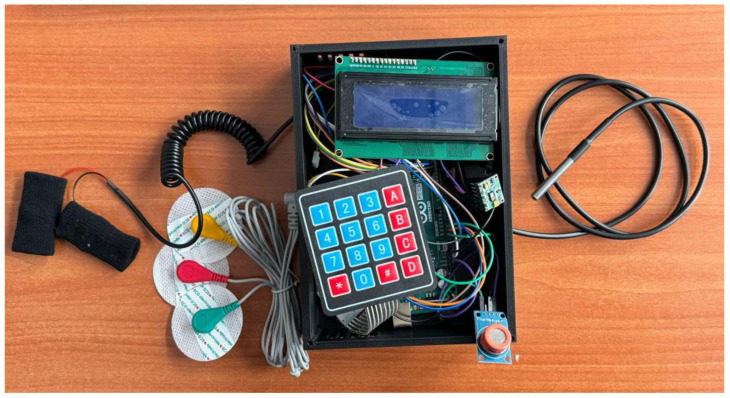
Internal structure and components the Medical Blackbox. The image shows the spatial arrangement of hardware components used in the Medical Blackbox, including the Arduino Mega board for multi-sensor signal acquisition, the ESP8266 module for wireless communication, and biomedical sensors for ECG and EMG monitoring. The power supply connection and sensor interface wiring are also visible, illustrating the system’s design for real-time physiological data acquisition and transmission within connected health monitoring scenarios.

The Medical Blackbox is a multi-sensor health monitoring device designed to measure key physiological parameters with high precision. The Medical Blackbox is designed as a stationary device intended for use in both clinical and home environments, where it can be deployed near the patient for real-time monitoring. While it is not wearable, the device’s form factor and low-power operation support relocation between settings, ensuring flexibility in remote healthcare scenarios. The device features an intuitive LCD display and a user-friendly keypad for selecting different monitoring modes, ensuring accessibility across various healthcare environments, from clinical use to home settings (Figure 7). Built on the Monit4Healthy architecture, it integrates biosensors previously outlined in this study, including those for ECG, GSR, and SpO_2_ measurements. Its design incorporates PPG sensors for enhanced heart rate and SpO_2_ accuracy, as well as EMG signal acquisition capabilities, which will be further discussed in subsequent sections. Figure 7 showcases a view of the Medical Blackbox, with its internal structure and components.

**Figure 8:**
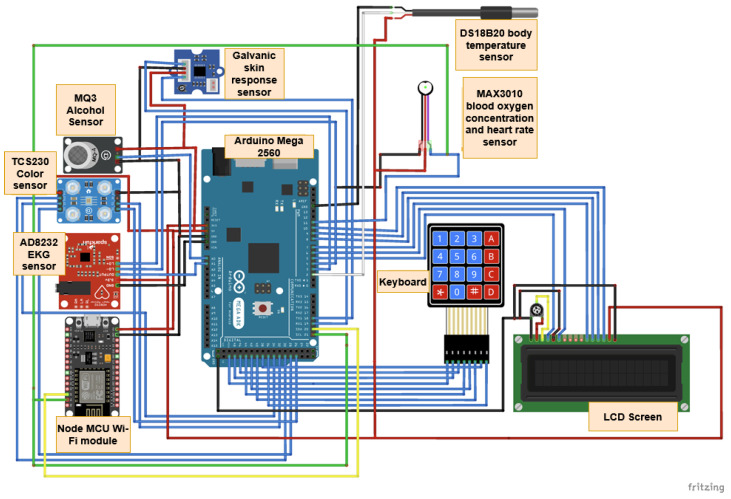
Electrical schematic of the Medical Blackbox, illustrating the integration of multiple biomedical sensors with the Arduino Mega microcontroller. The schematic was developed.

The integration of these sensors within the Medical Blackbox (Figure 8), in combination with the Arduino Mega microcontroller board, enables comprehensive and continuous data acquisition. Unlike other devices in the system that use low-power microcontrollers such as ESP32 and STM32, the Medical Blackbox employs an Arduino Mega, chosen for its versatile I/O capabilities and suitability for prototyping multi-sensor signal acquisition. To ensure high-fidelity signal capture, signal conditioning techniques—specifically filtering and amplification—are applied to the raw data streams, effectively enhancing signal quality and minimizing noise interference. The microcontroller unit (MCU) facilitates real-time processing by executing embedded algorithms that assess sensor outputs, enabling the detection of anomalies and supporting immediate alert generation and system responses. Data communication is established wirelessly via the NodeMCU ESP8266 module. The processed data are subsequently transmitted to a Raspberry Pi 4 Model B, functioning as a gateway device, which aggregates the data and forwards them to cloud-based infrastructures for advanced processing, storage, and visualization. Communication between the integrated sensors and the Arduino Mega is implemented using the I^2^C protocol—a widely adopted serial communication interface recognized for its efficiency and reliability, requiring only two conductors for data exchange.

The Medical Blackbox is powered through a regulated external adapter compatible with standard 220V outlets, ensuring the stable and safe operation of all internal components as illustrated in the electrical schematic.

#### 4.1.2. The EMG Blackbox Device

**Figure 9:**
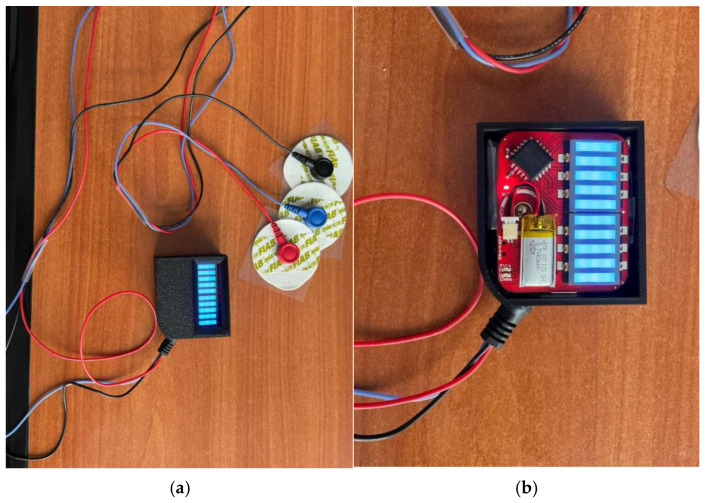
The EMG Blackbox: (**a**) frontal view showing the compact enclosure housing the data acquisition unit; (**b**) internal structure illustrating the integration of biomedical sensors, signal conditioning circuitry, and microcontroller components used for real-time EMG signal processing and wireless data transmission.

Geared toward monitoring patient neuromuscular activity, the EMG Blackbox employs high-sensitivity gel electrodes to record muscle electrical activity with minimal interference. To improve the accuracy of the EMG signal, the device incorporates noise-filtering techniques for eliminating artifacts. Preliminary laboratory tests indicate that the EMG Blackbox is highly effective in capturing muscle activity patterns with high precision and minimal latency, therefore allowing healthcare specialists to assess the evolution of muscle function, and detect early signs of neuromuscular deterioration, ensuring a proactive approach to patient monitoring. A frontal view of the device, as well as its internal structure, can be observed in Figure 9.

The EMG Blackbox device is designed for capturing and analyzing EMG signals in real time. It integrates multiple technological components to ensure the high-fidelity acquisition and wireless transmission of muscle activity data. The core sensing component consists of surface EMG electrodes, which record the electrical activity of the target muscles. The MyoWare 2.0 Wireless Shield processes the EMG signals, while the MyoWare 2.0 LED Shield visually indicates muscle contraction intensity. For wireless communication, the system employs the ESPRESSIF ESP32-WROOM-32E module, which facilitates secure Wi-Fi connectivity to the local network and platform. Power is supplied either via a USB-C interface or through a rechargeable battery, ensuring operational flexibility. The system is configured for straightforward deployment: the EMG sensors are attached to the specific muscle groups, and the ESP32 microcontroller is programmed to collect, process, and transmit the acquired signals. Data integration into the Monit4Healthy platform is accomplished via API configuration, enabling comprehensive clinical analysis and patient management based on EMG data.

**Figure 10:**
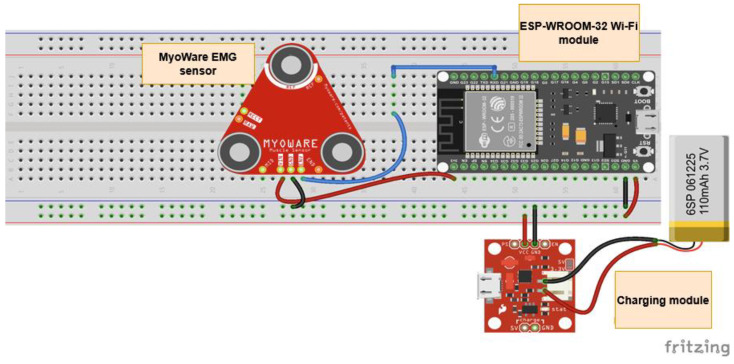
Electrical schematic of the EMG Blackbox device developed using Fritzing software. The schematic illustrates the integration of a MyoWare EMG sensor with the ESPRESSIF ESP32-WROOM-32E Wi-Fi module for wireless data transmission. Power supply is provided through a rechargeable 3.7V Li-Po battery, managed by a dedicated charging module.

The EMG Blackbox is built using off-the-shelf components, selected specifically for their reliability, accessibility, and ease of integration in prototyping and research environments. The device utilizes commercially available modules such as the MyoWare 2.0 EMG sensor and the ESPRESSIF ESP32-WROOM-32E Wi-Fi module, interconnected via standard breadboard configurations. Schematics (presented in Figure 10) were designed using Fritzing software 1.0.4, to clearly illustrate the hardware integration and signal flow. While the current implementation leverages off-the-shelf components to expedite development and testing, future iterations of the EMG Blackbox will focus on designing a custom PCB, which will allow for miniaturization, improved signal integrity, and enhanced suitability for clinical use cases.

### 4.2. Monit4Healthy Platform Development

The Monit4Healthy system continuously collects, processes, and visualizes real-time health data, permitting patients to monitor their vital signs, movement stability, and neuromuscular data through an interactive interface. Concomitantly, medical professionals can access detailed patient profiles to visualize historical data and analyze long-term health evolution, with the platform allowing clinicians to continuously monitor a patient and perform risk assessment. Automated alerts notify users of abnormal values or events, contributing to timely interventions.

#### 4.2.1. Monit4Healthy Platform Overview

The Monit4Healthy platform is designed to support two primary user roles—patients and medical professionals—each with specific, tailored functionalities. Following authentication, users are directed to their personalized dashboards, displaying real-time data. The patient dashboard presents the main health parameters of the patient, which include the pulse, SpO_2_, temperature, movement stability, and fall detection status, while the medical professional’s dashboard provides a detailed overview of patient data, allowing them to monitor patient health data and respond promptly to urgent events.

The platform allows the analysis of historical data and graphical representations of medical information, intended to offer a clearer visualization of the data, and ultimately aid in proactive risk management. The platform’s primary purpose is to integrate health information into a user-friendly interface, allowing for the monitoring of metrics, managing medical recommendations, and addressing potential health concerns.

The Monit4Healthy platform is designed with a Romanian-language interface, in order to support the accessibility of its primary users. To make navigation intuitive for both patients and healthcare professionals, all system functionalities, including dashboards, medical data visualizations, device management, and recommendations, are presented in Romanian. Throughout this section, the platform interface is illustrated alongside translated Romanian key terms to facilitate a better understanding of the interface.

#### 4.2.2. Patient Interface

The Monit4Healthy patient dashboard provides an overview of the most recent medical data collected, allowing the patient to be informed and engaged in their health management. The dashboard displays physiological and biomechanical data, within measuring dials, including “Puls (Pulse)”, “SpO_2_”, “Temperatură (Body Temperature)”, “Alcool Expirat (Alcohol Concentration)”, “Unghi Postural (Postural Angle)”, “Deplasare Laterală (Lateral Displacement)”, and “Detecție Cădere (Fall Detection)”. These parameters are continuously acquired from the medical devices and updated in real-time, therefore allowing patients to track changes in their vital signs and mobility status.

**Figure 11:**
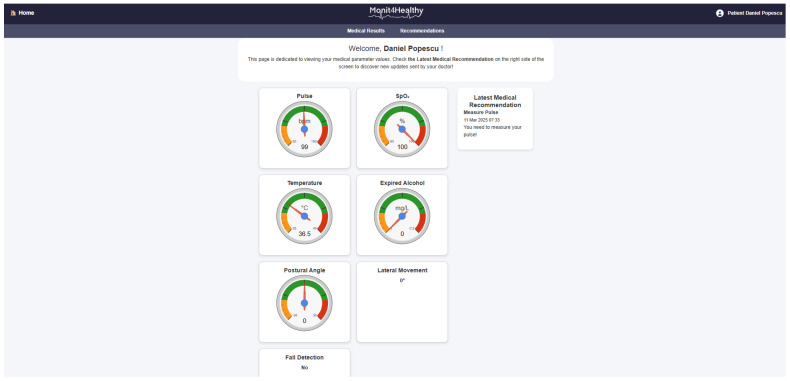
The Monit4Healthy patient dashboard. The dashboard interface designed for patient use provides real-time visualization of key health parameters acquired via the Monit4Healthy system. It displays physiological data such as ECG, EMG, heart rate, and temperature in both numeric and graphical formats, allowing users to track their current status and detect anomalies. The interface also includes trend graphs for historical data analysis and interactive alerts for significant deviations from normal values. This component ensures user-friendly access to health information, supporting timely awareness and personalized health management.

Additionally, as illustrated in Figure 11, to the right of the page, the dashboard displays “Ultima Recomandare Medicală”, which is the most recent medical recommendation provided by their assigned clinician. This functionality enables efficient communication between the healthcare specialist and the patients in their care, supporting adherence to medical advice.

**Figure 12:**
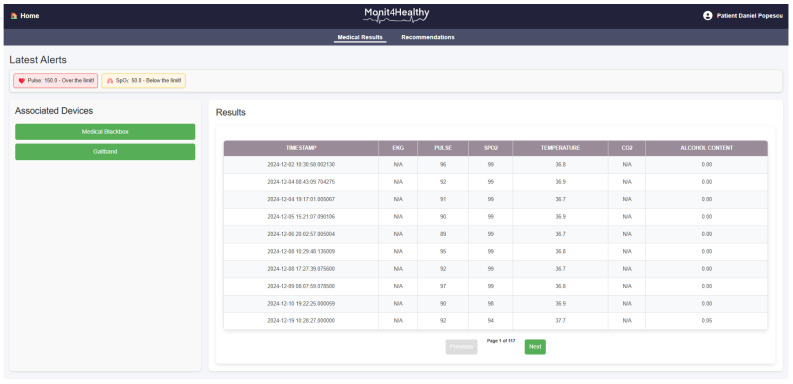
The Medical Results section in the patient’s platform interface. This section of the Monit4Healthy patient platform enables users to review detailed medical evaluations and historical health data. It consolidates diagnostic results, laboratory findings, and physician observations in a structured format. The interface supports secure access to medical documents, visual summaries of health trends, and export options for sharing data with healthcare professionals. This functionality enhances patient engagement and facilitates continuity of care through transparent access to individualized medical records.

Amidst the key sections of the platform are “Rezultate medicale (Medical Results)” and “Recomandări (Recommendations)”, both of which are designed to allow patient access to health information. The former is dedicated to providing a detailed view of the patient’s most recent medical alerts (“Ultimele Alerte”) and recorded physiological parameters (“Rezultate”), as well as a list of devices that have been assigned to them for data collection (“Dispozitive Asociate”). As shown in Figure 12, a detailed log of historical health data permits the patient to analyze detected anomalies over time, while the overview of medical devices assigned offers transparency regarding the sources of the collected data and reinforces the patient’s awareness of their monitoring regimen. The section offers a retrospective analysis of health trends, therefore offering patients the opportunity to identify patterns in their physiological data, potentially assisting in recognizing factors that contribute to fluctuations in their health status.

**Figure 13:**
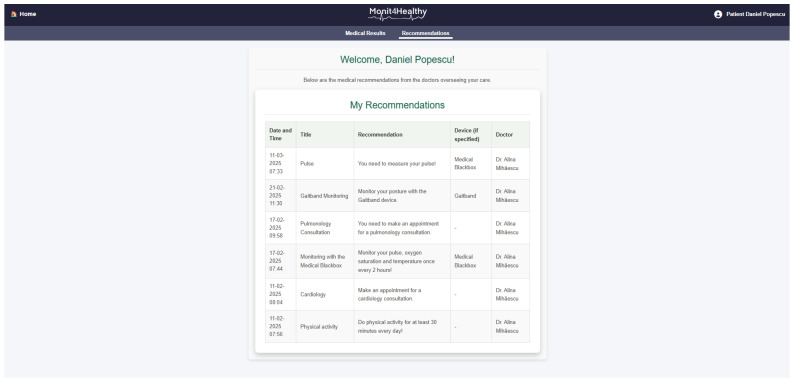
The Recommendations section in the patient platform interface. This section provides personalized health advice, lifestyle recommendations, and system-generated alerts based on the analysis of physiological data collected by Monit4Healthy devices. It offers users tailored guidance aimed at improving health outcomes, supporting self-management, and fostering proactive engagement in personal health monitoring.

The “Recomandări (Recommendations)” section provides a chronological view of all medical recommendations that the patient has previously received from their healthcare provider. The “Recomandările Mele (My Recommendations)” table maintains an organized history of all past clinical guidance, mentioning the date, a summary of the recommendation (“Titlu”), a detailed description of the clinical advice (“Recomandare”), the device with which the patient should monitor their vitals (“Dispozitiv, dacă este specificat”), and the medical professional that issued the recommendation. The section supports the patient in monitoring their own adherence over time and encourages patient compliance with prescribed recommendations. Thus, the platform ensures transparent access to medical data and contributes to a patient-centered approach to digital health monitoring. Figure 13 presents the “Recomandări” (Recommendations) page from the patient interface.

#### 4.2.3. Medical Professional Interface

Designed to facilitate real-time patient management and health monitoring, the Monit4Healthy clinician dashboard serves as a main control panel through which healthcare professionals can oversee patient data, manage device assignments, and promptly respond to sensor-triggered alerts issued by the devices within the Monit4Healthy system. The clinician dashboard provides an exhaustive overview of all monitored patients, ensuring that the medical professionals have instant access to patient health information.

A centralized table “Ultimii pacienți monitorizați (Last Monitored Patients)” presents the key patient details, including the first and last name of the patient, their e-mail, and personal identification code (“CNP”), alongside the assigned medical devices (“Dispozitive asociate (Associated Devices)”), offering a clear understanding of the monitoring capabilities available for each patient.

On the right panel of the interface, a series of statistics are displayed within cards to assist the clinician in resource allocation and patient monitoring.

**Figure 14:**
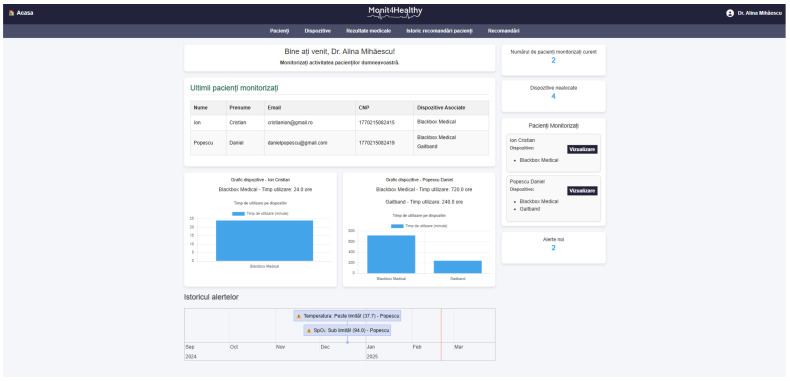
The Monit4Healthy medical professional dashboard. This dashboard provides healthcare professionals with real-time access to patient data, including vital signs, monitoring trends, and alert notifications. It enables efficient patient management through data visualization tools, customizable reports, and secure access to longitudinal health records, supporting informed clinical decision making and timely interventions.

The “Numărul de pacienți monitorizați curent (Number of currently monitored patients)”, “Dispozitive nealocate (Number of unassigned devices)”, “Pacienți Monitorizați (Monitored Patients)”, and “Alerte noi (New Alerts)” are meant for trend analysis in order to detect potential deteriorations in the patients’ conditions. As Figure 14 illustrates, at the bottom of the dashboard, the “Istoricul Alertelor (Alert History)” section provides a chronological view of past alerts, allowing medical professionals to track variations in patient health status.

**Figure 15:**
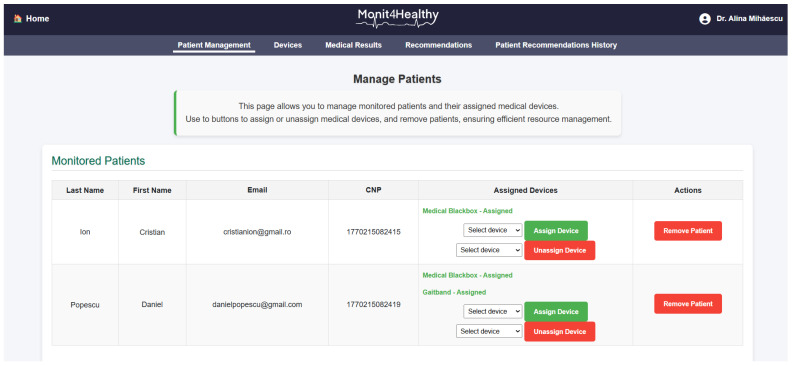
The Patients section from the medical professional’s interface. This section allows clinicians to view and manage their assigned patients, providing a summary of each patient’s monitoring status, recent alerts, and key health parameters. The interface supports efficient navigation through patient profiles, enabling streamlined access to individualized health data and historical records for continuous care management.

The Monit4Healthy platform offers an efficient patient management interface, allowing clinicians to associate and dissociate medical devices as needed. This section includes a structured table “Pacienți și Dispozitive (Patients and Devices)” that lists the patients and their associated devices, for the clear visualization of ongoing monitoring activities. Interactive features such as a dropdown menu “Alege dispozitiv (Choose a device)” and the “Atribuie Dispozitiv (Assign the device)” button allow the medical professional to select and assign an available medical device to a patient. Once a selection has been made, the platform ensures integration with the patient’s records. Similarly, if a device is no longer relevant for monitoring a certain patient’s health status, it can be dissociated from the patient profile, by pressing the “Șterge Dispozitiv (Remove device)” button, therefore allowing for the optimal distribution of the devices among the monitored patients. Additionally, the list of patients under the clinician’s care can be modified dynamically, providing flexibility for managing assigned patients, with the medical professionals being able to eliminate a patient with ”Șterge Pacient (Remove a Patient)” from their patient list. As shown in Figure 15, this section offers an overview of the assigned patients, therefore ensuring optimal device allocation and resource usage.

**Figure 16:**
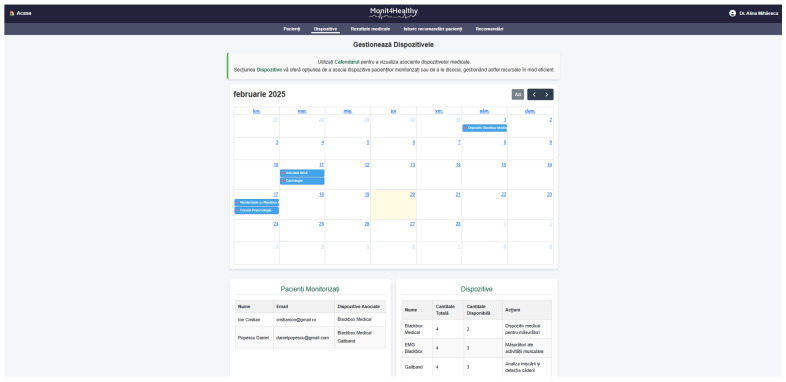
The Devices section from the medical professional’s interface. This section displays all active Monit4Healthy devices assigned to patients, including their operational status, device identifiers, and recent data transmission logs. Medical professionals can monitor the connectivity and functionality of each device in real time, ensuring seamless data acquisition and prompt identification of technical issues.

The Devices section provides an overview of medical device assignments, with an integrated calendar facilitating a clear visualization of assignment history. This functionality ensures that all device assignments are properly documented. The section includes two tables: “Pacienți Monitorizați (Monitored Patients)” detailing monitored patients along with their assigned devices, mapping the patients to the physiological parameters tracked; and “Dispozitive (Devices)”, acting as an inventory of all medical devices within the system. This offers medical professionals an overview of available resources and contributes to optimizing device allocations. The Devices page from the clinician’s interface can be viewed in Figure 16.

The Medical Results section of the Monit4Healthy platform serves as an interface for accessing and analyzing patient data collected from multiple connected devices. This section integrates real-time data visualization, filtering mechanisms, and customizable graphical representations, offering flexible access to medical professionals to patient metrics.

**Figure 17:**
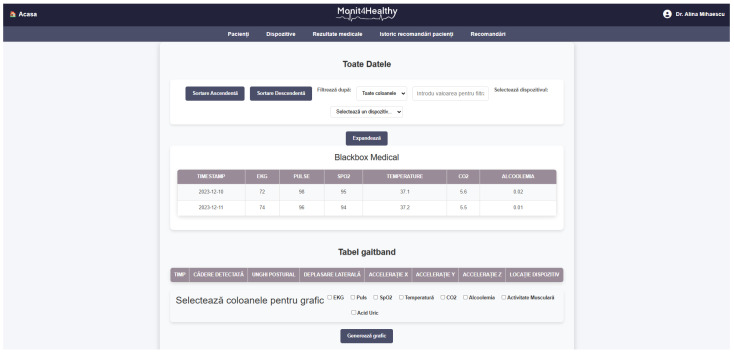
The Medical Results section from the medical professional’s interface. This section provides healthcare professionals with detailed visualizations of biomedical data collected from patients, including charts, anomaly indicators, and historical trends. It supports clinical decision making by offering real-time access to physiological parameters, ensuring comprehensive and efficient patient monitoring.

The interface offers a structured and easily navigable layout, with a left-side panel displaying a list of all monitored patients alongside their relevant information such as names, assigned medical devices, and available data records. Upon selecting a patient, the most relevant medical results are displayed, organized into structured tables, each corresponding to a different monitoring device, as illustrated in Figure 17. The system allows for a detailed examination of patient data, with timestamps ensuring a clear chronological perspective on health trends. To further strengthen accessibility, the platform includes sorting and filtering options, allowing clinicians to refine the dataset based on specific timeframes, or parameters of interest.

A core feature of this section is the ability to graphically represent medical data by clicking the “Generează grafic (Generate a graph)” button, providing a more intuitive way to interpret the evolution of patient health status. Users can select specific physiological parameters for visualization, generating dynamic graphs that highlight fluctuations over time, aiding in the early detection of health deterioration or improvement.

- The History of Medical Recommendations section of the Monit4Healthy platform is designed to facilitate the review of past clinical guidance provided to patients. This section provides an interactive interface incorporating a dropdown menu, allowing clinicians to select a specific patient. Upon selection, the platform dynamically displays all previously issued recommendations for the specific patient. This historical overview permits the medical professional to identify trends in patient response to recommendations.

The Recommendations section allows clinicians to issue new medical recommendations. To the left of the interface, the section features an input form where physicians are required to select a patient (“Selectează Pacient—Select a pacient”) and, optionally, an associated medical device (“Selectează Dispozitiv, opțional”—Select a device, optional), before submitting a new recommendation. On the right side of the interface, once a patient is selected, their clinical details are displayed, including the “Nume, Prenume, Vârstă (Patient first and last name and age)”, “Patologie (Associated pathologies)”, and “Ultimele date înregistrate (Most recently recorded data)”.

**Figure 18:**
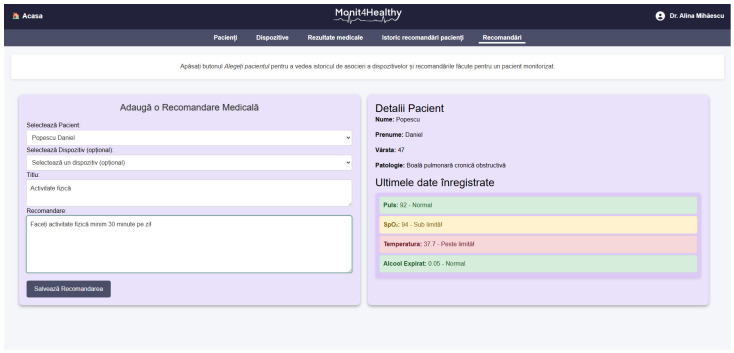
The Recommendations section from the medical professional’s interface. This section allows medical professionals to provide personalized health recommendations based on patients’ monitored data. It facilitates the creation, management, and communication of tailored advice, supporting proactive care and fostering continuous engagement between clinicians and patients.

As illustrated in Figure 18, the input form requires a “Title (Titlu)”, providing a summary of its purpose, and a dedicated section for detailed clinical guidance (“Recomandare”), specifying the parameters to monitor. This functionality guarantees that clinical advice is well documented, as well as systematically recorded, therefore assuring clinical workflow efficiency and improved patient engagement and adherence to medical advice.

#### 4.2.4. Real-Time Data Transmission and Alerts

A fundamental aspect of the Monit4Healthy platform is its real-time data acquisition and visualization, with continuous patient health metric monitoring. As soon as a connected medical device receives new information, the data are transmitted to the platform, processed, and presented on the user’s dashboard with minimal latency. To ensure patient safety, the system includes an alert mechanism to notify users of deviations from predefined physiological thresholds, with the system instantly generating visible alerts for both the patient and the physician.

#### 4.2.5. Platform Authentication and User Management

**Figure 19:**
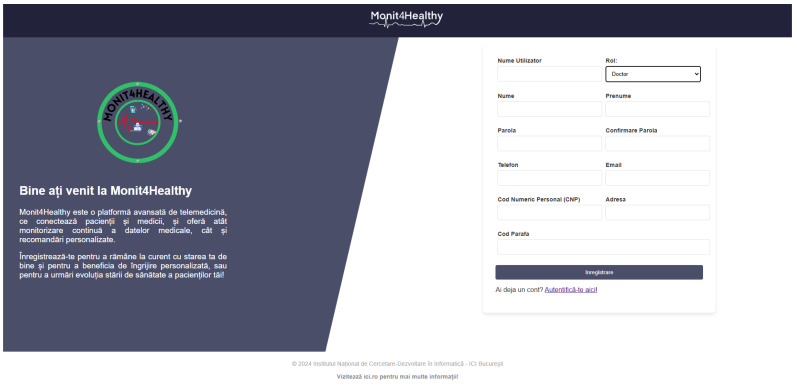
The Authentication page in the Monit4Healthy platform. This page ensures secure access to the platform through user authentication. It supports login for both patients and healthcare professionals, maintaining data confidentiality and system integrity by restricting access to authorized users only.

The Monit4Healthy platform utilizes a secure authentication framework to ensure role-based access control. The system differentiates between the two primary user roles, each granted distinct access permissions. During account creation, patients register by providing personal details, while medical professionals must provide a unique medical identification code (“*Cod Parafă*”) to verify their professional credentials. This verification step ensures that only licensed healthcare professionals can access patient data, therefore assuring platform security and regulatory compliance. Figure 19 showcases the Monit4Healthy system authentication page, with the selected medical professional role, in filling out the identification code in order to create a new account.

### 4.3. Development Phase Observations

Preliminary laboratory tests have indicated that the Monit4Healthy platform offers a user-friendly experience, being highly intuitive and with a structured, easily navigable interface for both patients and healthcare professionals. System responsiveness was assessed, with multiple devices transmitting data concurrently. The platform demonstrated minimal latency, with real-time updates appearing nearly instantly in the platform upon data acquisition.

The accuracy, transmission efficiency, and reliability of the sensor data acquisition was tested, with the medical devices demonstrating a robust integration within the platform, ensuring efficient data flow from the sensors to the cloud and to the user interface. With the use of Raspberry Pi as a gateway, the process of data transmission was optimized for minimal latency and bandwidth efficiency.

To ensure data accuracy in the Monit4Healthy system, all sensors used for biomedical signal acquisition—specifically ECG, EMG, PPG, GSR, and body temperature sensors—underwent calibration and validation procedures during laboratory testing. Calibration was conducted using reference measurements obtained from standardized commercial devices and controlled environmental conditions, with sensor outputs assessed to confirm compliance with tolerances specified for healthcare applications. Where applicable, factory calibration parameters were reviewed and verified. For example, the PPG sensor (MAX30102) used for heart rate and SpO_2_ monitoring was factory-calibrated and provided an SpO_2_ accuracy of ±2% (within a 70–100% range) and heart rate accuracy of ±3 BPM, as specified by the manufacturer. Similarly, the temperature sensor (DS18B20) offers an accuracy of ±0.5 °C within the range of −10 °C to +85 °C and was validated under laboratory conditions. For the ECG and EMG modules, signal accuracy was evaluated using laboratory-grade signal simulators with standardized output parameters. These simulators generated reference waveforms with amplitude ranges between 0.5 mV and 4 mV, enabling the verification of acquisition accuracy and confirming appropriate filtering for clinical relevance. Although the system is currently in the final development and validation phase, with laboratory testing confirming stable and reliable sensor performance, comprehensive clinical accuracy benchmarking against certified medical devices is scheduled as part of the real-world deployment. These activities are detailed in the implementation roadmap and will involve comparative evaluations under clinical conditions to confirm sensor precision and consistency in real-world scenarios.

As previously mentioned, among the evaluated aspects during the development phase was the performance and placement optimization of the Gaitband. While initially placed on the ankle, laboratory tests revealed that resting positions were frequently misinterpreted as falls. Upon detecting changes in orientation, such as leg repositioning or shifting weight, the accelerometer and gyroscope triggered false alerts. The most reliable and stable alternative was placing the device on the chest. This reduced false positive alerts and improved the accuracy of the balance assessment. GPS tracking was also incorporated in order to detect the location of the patient upon detecting a fall event.

As the system is still in its final stages of development, the experimental evaluation of the Monit4Healthy system so far has shown tangible gains in communication efficiency, confirming the effective application of structured data processing and communication techniques. The outcomes demonstrate that the system consistently gathers and processes biomedical signals while maintaining steady and dependable data exchange in a laboratory environment. Performance assessments further show that the system successfully reduces transmission inconsistencies, preserving an ongoing communication flow under various operational conditions. In enhancing the dependability and continuity of real-time data exchange, the Monit4Healthy system has the potential to overcome important challenges in connected health ecosystems, which is in line with the goals of next-generation IoT health monitoring systems.

A preliminary laboratory evaluation of the system’s reliability focused on evaluating fault tolerance, connection resilience, and signal consistency throughout extended usage. No significant issues were found over multiple continuous 8 h testing sessions, and under controlled conditions, the system maintained consistent wireless connectivity with a packet loss rate of less than 1%. Real-time data transmission was uninterrupted, and sensor signal integrity was maintained. Although these results show dependable short-term performance, further research to guarantee long-term dependability in various healthcare contexts will still need to include large-scale deployment measurements and prolonged runtime evaluations (such as the Mean Time Between Failures).

These findings demonstrate that the Monit4Healthy system, in its current laboratory configuration, achieves stable performance and efficient resource utilization under typical monitoring conditions, validating its readiness for further clinical validation and optimization.

## 5. Discussion

By incorporating multi-sensor fusion, real-time biomedical signal processing, and efficient data transmission approaches, the Monit4Healthy system enables a cohesive structure for IoT-enabled health monitoring. The system addresses challenges related to sensor interoperability, reliable information exchange, and real-time monitoring features in order to address meaningful challenges in IoT-driven healthcare. Its multi-layered architecture allows highly effective data gathering, edge processing, and secure communication protocols, providing accurate physiological and biomechanical data analysis. Its usefulness across connected health ecosystems is sustained by laboratory validation, which demonstrates its capacity to provide responsive signal processing, continuous monitoring, and comprehensive health data management.

*Main contributions and foreseen performance impact*

The Monit4Healthy system’s *structured data transmission process* has been designed as one of the most important factors that aims to decrease energy consumption and network congestion while improving real-time biomedical signal processing. Future assessments will be carried out to establish the extent of these expected improvements. Only relevant and processed health parameters are transmitted owing to the system’s edge-level data preprocessing, responsive data selection, and event-driven alert mechanism. It is a better alternative for typical IoT-based health monitoring systems as it successfully minimizes data redundancy while maintaining high data integrity and real-time responsiveness.

Preprocessing based on edge computing is a key element of this approach, since it considerably decreases the amount of raw health data sent to the cloud. In order to make sure that only the most relevant health parameters can be processed into optimum datasets, physiological signals collected through sensors like ECG, EMG, PPG, and GSR are subject to initial filtering, feature extraction, and aggregation at the data gathering stage. By reducing used bandwidth in the same time with preserving signal reliability, this process allows for smooth data transmission while not burdening computational resources.

The Monit4Healthy system updates its communication protocols according to network scenarios and the data level of importance to further enhance structured data transmission. Wi-Fi transmission is used for the high-resolution transmission of health data when mandatory, while BLE is applied for short-range, energy-efficient data sharing. In order to facilitate the real-time detection of abnormalities and multi-sensor interaction, the system likewise makes sure that delivered data are structured with timestamps, sensor identifiers, and abnormal flags.

The Monit4Healthy system’s event-driven trigger alert system, which evaluates measured parameters according to clinical relevance, is a prominent part of its structured transmission process. Instead of continuously transmitting gathered data, the system assesses patterns in medical standards and ultimately triggers an alert when particular limits are surpassed. For instance, typical transmission cycles are overridden by a persistently aberrant ECG rhythm, guaranteeing timely response. In contrast, data are aggregated and sent at predetermined intervals when physiological values remain within normal limits. This minimizes excessive network congestion while providing medical professionals with thorough and organized insights.

Maintaining low-latency, energy-efficient, and high-integrity transmission of data while preserving real-time responsiveness is a significant challenge in IoT-enabled health monitoring. The Monit4Healthy system was designed with a hierarchical communication approach that incorporates edge computing to minimize needless data transmission. In contrast with typical IoT health monitoring solutions that depend on constant raw data streaming to cloud-based infrastructures, this approach is expected to improve power efficiency and network resource allocation, and future evaluations will assess the performance of Monit4Healthy under real medical conditions.

Prior to being sent to an ICIPRO cloud-based server, collected data first go through edge-level filtering, feature extraction, and structuring. While maintaining data integrity and system responsiveness, this approach successfully lowers network congestion, maximizes bandwidth usage, and supports appropriate real-time monitoring.

The Monit4Healthy system’s fusion of edge and cloud computing is strengthened by the fact that edge-level processing reduces the need for constant raw data streaming to cloud servers by enabling real-time filtering, feature extraction, and data structuring. This approach significantly lowers the amount of data transmitted, which consequently decreases network congestion and improves reaction time for critical alerts compared to conventional cloud-only architectures. The system can handle time-sensitive tasks at the edges, like identifying anomalies and providing event-driven alerts, using a Raspberry Pi as a local processing unit. Solely necessary structured data for long-term storage and analysis are offloaded to the ICIPRO cloud infrastructure.

To illustrate the benefits of integrating edge computing in the Monit4Healthy system, a comparative analysis was conducted under laboratory conditions, focusing on latency and bandwidth usage in two configurations: edge–cloud hybrid (Monit4Healthy system) and cloud-only. In both scenarios, physiological data from ECG and PPG sensors were collected and processed. Edge-level preprocessing, performed on Raspberry Pi, reduced the volume of transmitted data by approximately 70%, as only filtered and feature-extracted data packets were sent to the cloud, compared to raw signal transmission in the cloud-only setup. For a 60 s monitoring session with ECG sampled at 250 Hz and PPG at 100 Hz (16-bit resolution), cloud-only transmission required approximately 0.040 MB per minute, while edge–cloud processing reduced this to around 0.012 MB/min. Latency measurements, defined as the time from data acquisition to cloud reception and processing, were reduced from an average of 350 ms (cloud-only) to 120 ms (edge–cloud), representing a 65% latency improvement. This efficiency supports scalable deployment in environments with constrained network resources and enables real-time responsiveness, crucial in health monitoring applications. While these results are preliminary and limited to controlled laboratory testing, they validate Monit4Healthy’s design objectives and establish a technical foundation for future benchmarking in clinical environments.

The Medical Blackbox device is powered by an external adapter in the current lab setting so that it can operate continuously. The proposed design takes into account the overall utilization of the active biomedical sensors, ESP8266 module, and Arduino Mega. Although these findings are suggestive, a thorough energy determination evaluation will be conducted to evaluate runtime variability in various operating scenarios.

A more thorough quantitative comparison between edge-enabled and cloud-only configurations—measuring specific metrics like transmission delay, data volume, and processing time—is planned in the system’s deployment roadmap, despite early laboratory evaluations confirming that edge computing contributes to lower latency and optimized bandwidth usage. In order to compare the system’s performance in real-life scenarios and to further assess the technological advantages brought about by the edge computing architecture, this future analysis will incorporate simulation-based and physical testing.

The Monit4Healthy system optimizes its flexibility by setting priorities and organizing the transmission of data at the edge level so that measured parameters undergo processing with the lowest possible level of latency. The system optimizes network use while keeping high-accuracy biomedical data collected by dynamically changing data transmission priorities based on sensor type and real-time health parameters. These features define the Monit4Healthy system as a scalable option for healthcare and remote patient monitoring solutions, wherein energy-efficient, low-latency functioning is required for the continuous tracking of the health status.

The Monit4Healthy system’s *multi-sensor fusion-based architecture* faces the drawbacks of single-sensor monitoring systems by enabling thorough health evaluations. The system increases the accuracy of diagnosis through linking many health information by means of the integration of ECG, EMG, PPG, and GSR sensors. Its flexible and modular architecture reinforces its application within connected healthcare ecosystems by facilitating adoption throughout a variety of healthcare environments, from clinical settings to home-based monitoring.

The Monit4Healthy system represents *an opportunity for integration with smart city infrastructures* beyond its main capabilities. The system may assist and provide support to digital health analytics platforms, emergency response coordination, and real-time health surveillance by integrating into already-existing urban IoT infrastructures. Large-scale urban health monitoring solutions may benefit from its scalable architecture and acquired data transmission structure through easy interoperability with healthcare services at the city level and AI-driven analytics, leading to the improvement of population-wide health assessment and resource management. In enabling predictive disease modeling and enhancing the use of healthcare resources, this potential integration fosters proactive health monitoring enlarged to the population level. The Monit4Healthy system seems to be appropriate for usage in assisted living environments, such as chronic disease management within smart healthcare regions, and elderly care, due to its secure data transmission capabilities and real-time biomedical sensor acquisition.

Although Monit4Healthy is primarily designed for remote and individualized health monitoring, the structured biomedical data it collects and processes can contribute to broader smart city healthcare ecosystems. In integrating anonymized health trends into municipal health platforms, the system can support data-driven decision making for resource allocation, urban health planning, and the early detection of community health risks. The potential interoperability of Monit4Healthy with digital health infrastructures enables its integration with emergency response systems, allowing healthcare providers to access real-time patient data when needed. This capability underlines the role of the Monit4Healthy system as a complementary component within connected health ecosystems, contributing to the development of more responsive and data-informed urban healthcare solutions.

To make sure that its sensor fusion approaches, data transmission protocols, and real-time signal processing features operate as designed under simulated healthcare circumstances, the Monit4Healthy system has undergone functional testing and validation in a controlled laboratory context. To verify that the system maintains high signal accuracy and reliable network transmission under various test situations, the testing process focused on assessing data reliability, sensor responsiveness, and connectivity stability. The system was evaluated using simulated physiological data inputs that emulated changing health monitoring circumstances in order to gauge real-time performance.

*Estimated performance contributions of the Monit4Healthy system*

With the goal of enhancing real-time biomedical monitoring, the Monit4Healthy system implements structured data transmission, edge computing abilities, and optimized sensor communication. The architectural design and laboratory testing and validation results highlight improvements in key performance aspects, such as latency reduction, bandwidth efficiency, and energy optimization, although formal large-scale benchmarking remains an area for future research.

- *Latency optimization*: The minimization of transmission delays is accomplished by employing local edge-level preprocessing, therefore reducing the volume of raw biomedical signals being delivered to cloud infrastructures. By substituting the mere reliance on cloud-based processing with real-time physiological filtering and feature extraction occurring at the acquisition level, the system ensures that it is clinically relevant and only transmits well structured data. This approach is expected to reduce latency in scenarios of high-priority health monitoring, such as in detecting arrhythmias based on ECG data, when event-triggered alerts are dire.
- *Bandwidth utilization efficiency:* The Monit4Healthy system implements adaptive data selection mechanisms, with the purpose of improving network efficiency. To reduce redundant data transmissions, the system replaces the approach of continuously streaming high-frequency signals with periodic reporting and event-triggered alerts. Given that network congestion and resource allocations need to be optimized when designing remote monitoring systems, this design is specifically efficient. The system’s dual-mode communication approach—using BLE for short-range sensor connectivity and Wi-Fi for higher-resolution data transmission when necessary—allows scalable and energy-efficient data exchange.
- *Energy consumption considerations:* The system improves energy efficiency by implementing a structured power management strategy, minimizing cloud reliance by passing computational tasks to local processing units. To optimize sensor-device interactions, the system utilizes BLE-based communications protocols, which have established low-power characteristics. Further quantitative analysis is required in order to validate the impact of these features in continuous monitoring scenarios, although they are anticipated to enhance energy-efficient operations. The reduction in continuous high-power transmissions, combined with the event-driven adaptive communication is anticipated to lengthen the lifespan of wearable and portable monitoring units. Despite these projections, battery consumption metrics need prolonged real-world evaluation. To additionally improve energy efficiency in extended health monitoring scenarios, future research could focus on duty-cycling mechanisms and machine learning-driven power scheduling.

*Limitations and challenges:*

- Real-world clinical assessments are still unexplored, despite laboratory testing confirming the system’s fundamental functioning. Future research is required to evaluate the Monit4Healthy system’s effectiveness in dynamic real-world settings, especially where network unpredictability, patient mobility, and telemedicine integration are involved.
- Another challenge is scalability, especially when it comes to easy integration with current telemedicine solutions and healthcare IT infrastructures. Even though the system’s modular architecture allows for flexibility, further testing is necessary to guarantee that it can handle extensive medical applications.
- Measurements for the long-term power usage of continuous monitoring have not yet been assessed apart from controlled laboratory settings. Although edge computing decreases redundant data transmission, energy efficiency is expected to be boosted using adaptive power management approaches, especially for wearable and portable applications; future experimental validations need to be performed to identify the practical advantages of these approaches in real-word settings.

*Future work* involves further research in several important directions in order to improve the Monit4Healthy system’s practical relevance:

- Carrying out extensive experiments to assess system performance in genuine clinical and digital health settings.
- Implementing a full English language option to improve usability for international users.
- Connecting EHRs and other type of healthcare solutions to provide simple interoperability and data sharing.
- Investigating hybrid IoT communication models that provide high-bandwidth, low-latency medical data transmission by integrating 5G-enabled frameworks.
- Future developments focusing on implementing advanced multi-sensor fusion algorithms, enabling the generation of composite health indicators by correlating synchronized ECG, EMG, PPG, and GSR data.
- Developing predictive analytics powered by AI to improve the automated identification of abnormalities and individualized health monitoring.
- To optimize network efficiency, current implementations are targeted toward structured data transmission. Despite ongoing approaches, future research could employ adaptive power management techniques, such as dynamic sensor sampling rate adjustments, to allow the system to reduce data acquisition frequency, and duty-cycling mechanisms, to power down non-essential components during periods of inactivity. To further lengthen the battery life, energy-aware task scheduling could be explored as well.
- Future experimental validations will quantitatively analyze Monit4Healthy’s energy consumption across various operating conditions, assessing the real impact of edge computing and adaptive data transmission on power efficiency.
- Integration with IoT-specific protocols like MQTT is being considered for future development phases, particularly to improve scalability and interoperability in smart city ecosystems or more intricate, dispersed healthcare infrastructures. This will further align the Monit4Healthy system with IoT communication standards by enabling efficient device-to-device and device-to-cloud connection.
- The potential benefits of edge computing will be addressed in more depth. This consists of real-world and simulation-based comparisons of edge-enabled and cloud-only setups, with an emphasis on important performance indicators including power consumption, processing time, data volume, and transmission latency. The findings from these investigations will allow for the additional optimization of the Monit4Healthy system’s edge-cloud integration strategy and quantitatively validate the architectural benefits of the system across various healthcare situations.
- Comprehensive clinical accuracy benchmarking against certified medical equipment is scheduled during real-world deployment phases to validate sensor performance under operational healthcare conditions.
- Adaptive communication strategies that dynamically optimize protocol selection and channel allocation in real time, aiming to enhance transmission reliability and energy efficiency in high-density and variable network environments, will be taken into consideration.
- Miniaturizing the Medical Blackbox by designing a custom PCB to replace the current prototyping board aims to optimize the device’s size, improve power efficiency, and enhance hardware reliability for long-term deployment in both clinical and home-based settings.
- Future research will involve thorough power profiling under a range of operating situations, such as continuous monitoring, event-driven alerts, and inactivity states, in order to comprehensively assess the energy efficiency of the Monit4Healthy system. All system components’ current drawing and consumption trends will be tracked using standardized energy measuring instruments. We will analyze metrics like overall battery duration under particular tasks, energy per transmitted data unit (mWh/MB), and average energy consumption (mW). These parameters will corroborate the system’s energy optimization assertions and direct future hardware and software improvements by enabling comparisons with other IoT-based health monitoring solutions. Future analyses will measure energy consumption per unit of processed biomedical data (e.g., milliwatts per kilobyte transmitted) and energy per monitoring session (e.g., Wh per 1 h operation under standard use settings) in order to assess the system’s energy efficiency. The baseline measurements from cloud-only monitoring systems with comparable sensor configurations but no edge-level preprocessing or structured data transfer will be compared to these measurements. The energy-saving effects of the Monit4Healthy system’s edge computing and adaptive transmission approaches will be measurable through this comparison.

Through the implementation of these improvements, the Monit4Healthy system will become more fitting for long-term deployment in remote health monitoring and wearable medical devices, thus guaranteeing sustained operation in resource-constrained environments.

*Roadmap for real-world deployment*

To ensure a structured transition of the Monit4Healthy system from laboratory validation to real-world healthcare environments, the following roadmap outlines the key phases of deployment, each with specific objectives, methodologies, and performance targets:

- Phase 1—Pilot Trials in Controlled Clinical Settings
  – *Objective*: Evaluate reliability, usability, and accuracy under supervision of medical professionals.
  – *Activities*: Deploy Monit4Healthy units in medical units for short-term trials; monitor ECG, EMG, and PPG signal acquisition; assess edge–cloud performance in operational conditions.
  – *Metrics*: User feedback, real-time alert responsiveness, signal integrity, data transmission stability, and power consumption profiles.
  – *Duration*: 3–6 months.
  – *Deliverables*: Technical report on system performance; validated integration with EHR platforms and telemedicine systems.
- Phase 2—Multi-Site and Home-Based Testing
  – *Objective*: Assess scalability, adaptability, and long-term reliability in diverse healthcare scenarios.
  – *Activities*: Deploy in multiple sites including home-care environments; focus on patient mobility, variable network conditions, and extended monitoring periods.
  – *Metrics*: Energy consumption trends, battery life in real conditions, network adaptability, anomaly detection rates, data privacy compliance.
  – *Duration*: 6–9 months.
  – *Deliverables*: Comparative performance analysis vs. cloud-only systems; refinement of adaptive power management; user satisfaction studies.
- Phase 3—System Optimization and Hardware Refinement
  – *Objective*: Miniaturize hardware components, improve energy efficiency, and enhance user ergonomics.
  – *Activities*: Design and fabricate custom PCBs for Medical Blackbox and EMG devices; integrate energy-aware scheduling; implement secure boot and firmware updates.
  – *Metrics*: Reduction in form factor, increased battery efficiency (target >12 h), and hardware validation.
  – *Duration*: 6 months (overlapping with Phase 2).
  – Deliverables: Optimized prototypes; industrial design for wearable/portable use; regulatory compliance planning (e.g., CE, FDA).
- Phase 4—Integration into Smart Health Ecosystems
  – *Objective*: Align Monit4Healthy system with smart city infrastructure.
  – *Activities*: Pilot integration with municipal health dashboards, emergency services, and AI-powered health trend analytics; implementation of MQTT for scalable IoT communication.
  – *Metrics*: Interoperability benchmarks, real-time data aggregation at population level.
  – *Duration*: 9–12 months.
  – *Deliverables*: Full-scale deployment proposal; partnership reports; scalability studies.

Together, these perspectives and planned developments position the Monit4Healthy system as a scalable, adaptive, and forward-looking solution, capable of enhancing real-time health monitoring and advancing the integration of IoT technologies in connected healthcare environments, including future deployment within smart city infrastructures.

## 6. Conclusions

Through the integration of multi-sensor fusion, IoT-enabled monitoring technologies, and appropriate data transmission frameworks, the Monit4Healthy system enables connected health ecosystems. The system tackles important issues in IoT-driven healthcare by enhancing sensor interoperability, real-time health monitoring, and secure data exchange. This allows for effective, scalable, and flexible health data processing.

The Monit4Healthy system improves biomedical signal integrity, network performance, and energy efficiency using edge computing and the structured transmission of information approaches. Thorough health evaluations are supported by its modular sensor fusion architecture, which enables easy integration into clinical, telemedicine, and smart city infrastructures. By means of the flexible prioritization of real-time health data and the decrease in needless network loads, the system promises high-reliability, low-latency medical monitoring. By enabling interoperability with digital health solutions, and the potential integration into smart city infrastructures, the Monit4Healthy system brings an opportunity to extend connected health ecosystems beyond conventional healthcare environments. To further consolidate its standing as a next-generation IoT-based health monitoring solution, future work and research will be centered around scalability, AI-driven analytics, and expanded implementations in current healthcare environments.

## Data Availability

Data are contained within the article.

## Abbreviations

The following abbreviations are used in this manuscript:

AFH	Adaptive Frequency Hopping;
AI	Artificial Intelligence;
BLE	Bluetooth Low Energy;
CAD	Computer-Aided Design;
CLI	Command Line Interface;
ECG	Electrocardiography;
EMG	Electromyography;
FHIR	Fast Healthcare Interoperability Resources;
GDPR	General Data Protection Regulation;
GPS	Global Positioning System;
GSR	Galvanic Skin Response;
HIPAA	Health Insurance Portability and Accountability Act;
I^2^C	Inter-Integrated Circuit;
IaaS	Infrastructure as a Service;
IoT	Internet of Things;
LoRaWAN	Long-Range Wide-Area Network;
ORM	Object Relational Mapper;
MCU	Microcontroller Unit;
PPG	Photoplethysmogram;
RPM	Remote Patient Monitoring;
SpO_2_	Oxygen Saturation;
SQL	Structured Query Language;
SSL	Secure Sockets Layer;
TLS	Transport Layer Security;
VM	Virtual Machine;
Wi-Fi	Wireless Fidelity.
